# Can genetic rescue help save Arabia's last big cat?

**DOI:** 10.1111/eva.13701

**Published:** 2024-05-23

**Authors:** Hadi Al Hikmani, Cock van Oosterhout, Thomas Birley, Jim Labisko, Hazel A. Jackson, Andrew Spalton, Simon Tollington, Jim J. Groombridge

**Affiliations:** ^1^ Durrell Institute of Conservation and Ecology, School of Anthropology and Conservation, Division of Human and Social Sciences University of Kent Canterbury Kent UK; ^2^ Office for Conservation of the Environment Diwan of Royal Court Muscat Oman; ^3^ The Royal Commission for AlUla AlUla Saudi Arabia; ^4^ School of Environmental Sciences University of East Anglia, Norwich Research Park Norwich UK; ^5^ Centre for Biodiversity and Environment Research, Research Department of Genetics, Evolution and Environment University College London London UK; ^6^ Island Biodiversity and Conservation Centre University of Seychelles Victoria Seychelles; ^7^ Department of Life Sciences The Natural History Museum London UK; ^8^ School of Animal Rural and Environmental Sciences Nottingham Trent University Nottingham UK

**Keywords:** Arabian leopard, camera traps, endangered species, genetic diversity, genetic rescue, noninvasive sampling, *Panthera pardus nimr*, small populations

## Abstract

Genetic diversity underpins evolutionary potential that is essential for the long‐term viability of wildlife populations. Captive populations harbor genetic diversity potentially lost in the wild, which could be valuable for release programs and genetic rescue. The Critically Endangered Arabian leopard (*Panthera pardus nimr*) has disappeared from most of its former range across the Arabian Peninsula, with fewer than 120 individuals left in the wild, and an additional 64 leopards in captivity. We (i) examine genetic diversity in the wild and captive populations to identify global patterns of genetic diversity and structure; (ii) estimate the size of the remaining leopard population across the Dhofar mountains of Oman using spatially explicit capture–recapture models on DNA and camera trap data, and (iii) explore the impact of genetic rescue using three complementary computer modeling approaches. We estimated a population size of 51 (95% CI 32–79) in the Dhofar mountains and found that 8 out of 25 microsatellite alleles present in eight loci in captive leopards were undetected in the wild. This includes two alleles present only in captive founders known to have been wild‐sourced from Yemen, which suggests that this captive population represents an important source for genetic rescue. We then assessed the benefits of reintroducing novel genetic diversity into the wild population as well as the risks of elevating the genetic load through the release of captive‐bred individuals. Simulations indicate that genetic rescue can improve the long‐term viability of the wild population by reducing its genetic load and realized load. The model also suggests that the genetic load has been partly purged in the captive population, potentially making it a valuable source population for genetic rescue. However, the greater loss of its genetic diversity could exacerbate genomic erosion of the wild population during a rescue program, and these risks and benefits should be carefully evaluated. An important next step in the recovery of the Arabian leopard is to empirically validate these conclusions, implement and monitor a genomics‐informed management plan, and optimize a strategy for genetic rescue as a tool to recover Arabia's last big cat.

## 1 INTRODUCTION

Genetic diversity underpins evolutionary potential and is essential for the long‐term viability of wildlife populations, many of which find themselves in rapidly changing environments driven by anthropogenic activity (Frankham et al., 2017). Unable to keep pace with the speed of habitat fragmentation, enforced allopatry elevates the risk of population bottlenecks, the deleterious effects of inbreeding, loss of genetic diversity, Allee effects, and reduced evolutionary potential (Allendorf et al., 2013). Such impacts can depress individual‐ and population‐level fitness, increase mortality, and elevate disease susceptibility (Keller & Waller, 2002; Smallbone et al., 2016). Pressures on small and/or declining populations are exacerbated by the ongoing and increasing effects of climate change, and they are often compounded by competition with human populations for limited or diminishing resources, leading to direct persecution (Abrahms, 2021). Such a perfect storm of negative drivers forces fragmented populations, and ultimately species, onto an extinction trajectory (Abrahms, 2021). Among mammals, these challenges are highlighted by the decline of large carnivores, felids in particular, such as the Florida panther (*Puma concolor couguar*; Johnson et al., 2010; Rodgers & Pienaar, 2018; Van De Kerk et al., 2019) and the cheetah (*Acinonyx jubatus*; Terrell et al., 2016; Weise et al., 2017).

Conservation managers tasked with species recovery need to determine the extent of genetic diversity that persists both within the threatened wild population and captive populations. Genetic rescue aims to (re‐)introduce novel alleles into a genetically impoverished population, potentially alleviating inbreeding depression and increasing population viability. Genetic rescue can also reduce the realized load of homozygous mutations by making these loci heterozygous. When a population declines, inbreeding and genetic drift increase homozygosity, and this changes the composition of the genetic load (Bertorelle et al., 2022; Dussex et al., 2023). Recessive deleterious mutations that were previously masked in the heterozygous state in the ancestral population become expressed in homozygous loci during population size decline. This effectively converts the masked load into a realized load, resulting in a reduction in individual fitness and population viability (Bertorelle et al., 2022). Recent reports of successful population recovery and their integration into conservation planning show the value of captive populations for genetic rescue (Fitzpatrick et al., 2020, 2023; Hoffmann et al., 2021; Jackson et al., 2022; Krojerová‐Prokešová et al., 2023; Miller et al., 2020). However, obtaining relevant data that can inform genetic rescue continues to be challenging, particularly for large carnivores that naturally persist at low densities (Alexander et al., 2015; Bellemain et al., 2007; Farhadinia et al., 2018; Jiang et al., 2015).

**FIGURE 1:**
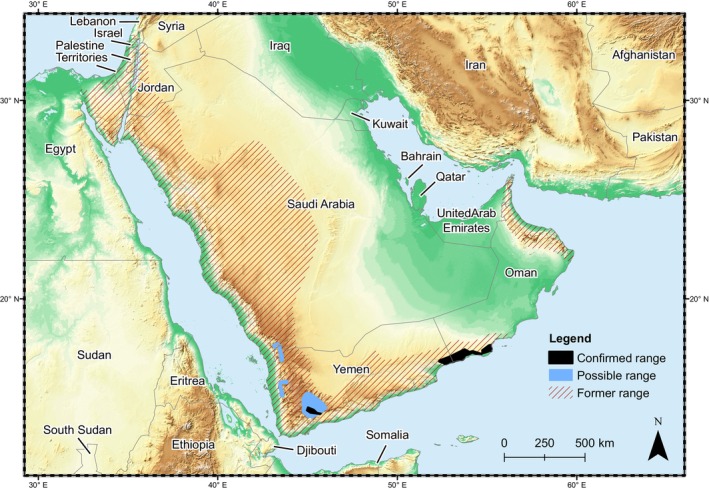
Historical and current distribution of Arabian leopards in the Arabian Peninsula.

**FIGURE 2:**
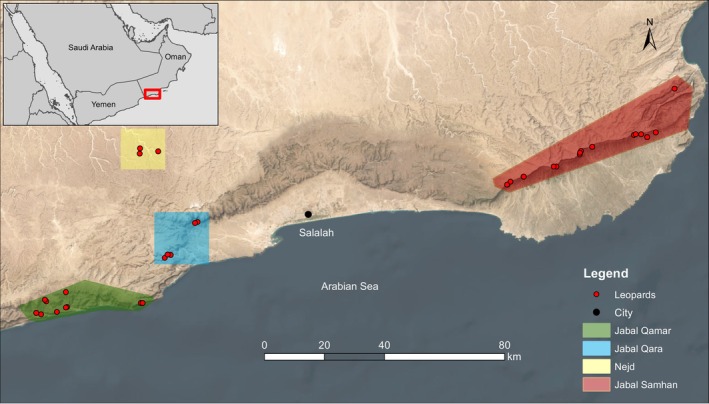
The location of the study regions in Dhofar and the spatial distribution of 36 leopards were identified from genetic analysis in this study. The inset map shows the location of Dhofar within Oman.

The Critically Endangered Arabian leopard (*Panthera pardus nimr*) is endemic to the Arabian Peninsula (Al Hikmani & Spalton, 2023). This highly elusive subspecies is differentiated morphologically from other *P*. *pardus* subspecies by its pale coloration and smaller body size, and through molecular phylogenetic analysis (Khorozyan et al., 2006; Mochales‐Riaño et al., 2023; Uphyrkina et al., 2001). The Arabian leopard was historically found across the Arabian Peninsula but has disappeared from most of its former range (Figure 1; Harrison & Bates, 1991; Breitenmoser et al., 2006). Today it is present only in small and fragmented populations in southern Oman and Yemen, with perhaps several individuals in Saudi Arabia (Al Hikmani et al., 2023). The most recent wild global estimate for the Arabian leopard is fewer than 100–120 individuals (Al Hikmani et al., 2023). In Oman, a population of 44–58 leopards is estimated to inhabit the Dhofar mountains in the south of the country (Spalton & Al Hikmani, 2014). Distributed across the three contiguous mountain massifs of Jabal Samhan, Jabal Qara, and Jabal Qamar and spanning approximately 250 km, this area is considered the last stronghold for the Arabian leopard (Figure 2; Breitenmoser et al., 2010). The 2013 discovery of several individuals in the Nejd—the northern foothills of Jabal Qara—represented a small northward extension of the current known range (Al Hikmani et al., 2015). Threats faced by Arabian leopards in the Arabian Peninsula include illegal killing by livestock owners, prey depletion, loss of prime habitat, and capture for the illegal pet trade (Al Jumaily et al., 2006; Spalton, Al Hikmani, Jahdhami, et al., 2006; Zafar‐ul Islam et al., 2018). Killing leopards in response to livestock depredation is currently considered the main cause of decline; at least 80 leopards were reported killed by local shepherds in Yemen, Saudi Arabia and the Musandam mountains of northern Oman between 1960 and 2023 (Al Hikmani & Spalton, 2023; Al Jumaily et al., 2006; Mensoor, 2023; Spalton, Al Hikmani, Jahdhami, et al., 2006; Zafar‐ul Islam et al., 2018).

Conservation initiatives for the Arabian leopard began in the mid‐1980s when the world's first captive breeding population was established in Oman from four wild leopards captured in Jabal Samhan. In the 1990s further groups of captive leopards—mostly wild‐caught individuals from Yemen—were established at centers in Yemen, United Arab Emirates, and Saudi Arabia. Some of these leopards were bred with the offspring of the original wild‐caught Omani leopards to retain the genetic diversity found in the wild population. By 2023, there were some 64 Arabian leopards in three facilities in the region (34 Sharjah, UAE; 27 Taif, KSA; 3 Oman, A. Alenzy, pers. comm. 2024), consisting of at least 14 founders (Budd & Leus, 2011). While additional captive individuals may exist in Yemen (Sanna and Taiz zoos) their numbers are unknown. The captive leopard population therefore holds great potential as a resource for future genetic rescue initiatives. Generating data on the patterns of distribution of genetic diversity across the wild and captive populations, alongside an accurate population‐size estimate for the wild population, are therefore critical.

Spatially Explicit Capture–Recapture (SECR) models can generate robust estimates of population density (Borchers & Efford, 2008) and have been applied to several big cat taxa (e.g., tiger *Panthera tigris* Kalle et al., 2011; Aziz et al., 2017; jaguar *Panthera onca* Sollmann et al., 2013) including leopards (Morris et al., 2022; Rahman et al., 2018; Vitkalova et al., 2018). Although SECR requires multiple recaptures to deliver accurate estimates—a challenging prerequisite for studies on highly elusive, wide‐ranging taxa—sampling DNA from fecal material (scats) can provide a noninvasive solution to this limitation and has been employed successfully to monitor tigers (*Panthera tigris tigris* Thapa et al., 2018), Snow leopards (*Panthera uncia* Janečka et al., 2008) and Amur leopards (*Panthera pardus orientalis*; Sugimoto et al., 2014). Used in combination, fecal DNA sampling and camera trapping can improve detection probability for more robust density estimates, authenticate density estimates derived from single methods, and provide a noninvasive approach to estimate population size and density. Combined, these approaches can inform how genetic diversity is distributed across populations of elusive carnivores (Dou et al., 2016; Gopalaswamy et al., 2012).

The Arabian leopard is the best‐known flagship species of the Arabian Peninsula. It has considerable environmental and cultural value, and its conservation remains a top priority for a variety of stakeholders across the region. Here, we use microsatellite DNA markers to provide the first comprehensive survey of genetic diversity and structure of the Arabian leopard across the wild and captive populations, provide a robust estimate of the wild population supported by SECR using both genetic data and camera‐trap data, and model the effects of genetic rescue on neutral genetic diversity, the genetic load, and population viability of the wild population.

## 2 METHODS

### 2.1 Sample collection

Between 2012 and 2017, scat collection surveys across the Dhofar mountains of southern Oman provided a total of 477 putative leopard scats. This material was augmented by 53 samples (scat, skin, blood) from wild and captive Arabian leopards of known provenance (including museum specimens) spanning the years 1976–2017. Complete sampling data are detailed in Table S1. Samples were transported to the UK for genetic analyses from Oman under CITES export permits 94‐97/2016 and import permits 548814/01‐2, and CITES export permit I7MEW2193 and import permits 555705/01‐3 from the United Arab Emirates.

### 2.2 DNA extraction and species identification using mtDNA

Genomic DNA was isolated from scat, skin, and blood samples using the QIAamp Fast DNA stool mini kit and the Qiagen DNeasy Blood and Tissue Kit (Qiagen, UK), respectively (see Supplementary Information for detailed methods S1). To identify, and subsequently exclude from further analyses, any scats derived from nontarget species (e.g., Arabian wolf *Canis lupus arabs*, striped hyena *Hyaena hyaena*, and caracal *Caracal caracal*), we amplified a 200 bp fragment of the NADH5 mitochondrial gene using leopard specific PCR primers (Uphyrkina et al., 2001) for all scats found. See Supplementary Information for full details of methods S1 for species identification.

### 2.3 Microsatellite amplification

We identified a set of 65 published polymorphic markers (Menotti‐Raymond et al., 1999; Mondol et al., 2009; Uphyrkina et al., 2001; Williamson et al., 2002) and tested their amplification success and extent of polymorphism in Arabian leopards using DNA from three scat samples genetically confirmed to be from leopards in Dhofar. Thirty‐five markers were successfully amplified during initial PCR trials and were then included in the design of seven multiplex sets (Table S2). Multiplex PCRs were performed using fluoro‐labeled forward primers to genotype all genetically confirmed leopard samples. Felid‐specific PCR primers designed to amplify the amelogenin and zinc‐finger regions of y‐chromosome were used to assign individual sex (Pilgrim et al., 2005). All PCR products were genotyped using an Applied Biosystems 3730 DNA Analyser and ROX 500 ROX™ size standard (DBS Genomics, Durham UK). We used Genemapper v3.7 (Applied Biosystems, UK) to identify and score the alleles. See Supplementary Information for full details on assessment of genotypes.

### 2.4 Identification of individuals from scat genotypes

Using samples from three known captive siblings, we used GIMLET to determine the probability of identity (PID) for siblings (sibs), and the minimum number of loci needed to distinguish between close relatives (Valiere, 2002). To identify 100% matched genotypes with the genotype data set, consensus genotype profiles were compared using Cervus v3.0 (Marshall et al., 1998). The sexing locus (AM; Pilgrim et al., 2005) was used as an additional means to verify the identification of duplicated samples of the same individual, with matched genotypes considered “recaptures” for the purposes of SECR (for full details see Supplementary Information).

### 2.5 Assessment of genetic diversity

#### 2.5.1 Objective 1: Identify global patterns of genetic diversity

To meet this objective, we partitioned the genotype data set for two analyses:

- *Comparisons of genetic diversity between populations of captive and wild individuals*. We made comparisons between all genotypes of wild Arabian leopards from Oman with those of captive Arabian leopards from breeding centers in Oman, UAE, and Saudi Arabia, including wild‐born leopards known to have originated from Yemen (see Table S3).
- *Comparisons of genetic diversity between wild Oman and wild Yemen leopards*. We partitioned the data into samples of wild‐born Arabian leopards from Yemen, and samples of wild Arabian leopards from Oman. Although samples of wild‐born Arabian leopards from Yemen are within the captive population of Arabian leopards held in Oman, UAE, and KSA, they are representative of the wild Arabian leopard population in Yemen. This partition thereby allows comparison of genetic diversity between the wild populations in Yemen and Oman.

#### 2.5.2 Objective 2: Quantify spatiotemporal patterns of genetic diversity

Given the extreme topography that delineates the different Dhofar mountain ranges, we compared genetic diversity between these different regions within Oman; wild Oman leopard samples were grouped according to the four sampling regions of Jabal Samhan, Jabal Qara, Jabal Qamar, and the Nejd (Figure 2).

- *Measuring the extent of genetic differentiation between different regions within Oman*. For analyses of genetic structure, we used a set of seven markers (FCA90, FCA105, FCA126, FCA279, 6HDZ89, 6HDZ635, 6HDZ700) that were polymorphic for the Dhofar population (36 leopards from four regions). GenAlEx v6.5 was used to quantify the extent of spatial genetic differentiation (*F*_ST_) between populations and to test for isolation by distance (IBD) using the Mantel test (Mantel, 1967). We also performed a spatial autocorrelation analysis using GenAlEx v6.5. Full details can be found in Supplementary Information. We used GENELAND to examine signals of genetic structure within the data set comprising leopards from the Dhofar mountain ranges, following two steps as per Guillot, Mortier, and Estoup (2005). GENELAND uses individual multi‐locus genotypes together with their geographic locations to infer the number of populations and identify any genetic discontinuity within these populations (Guillot, Estoup, et al., 2005). GENELAND analysis is considered to provide superior estimates of the number of clusters as it takes account of the geographic location of each sample, and is considered more robust in instances where there is a relatively weak genetic structure (Basto et al., 2016). We first applied 10 independent runs with 500,000 MCMC iterations and a burn‐in of 100 under the spatial model, specifying uncorrelated allele frequency assuming unknown *K*. To generate a map of the distribution of each cluster and accurate individual assignment, we repeated the analysis but treated the number of clusters as known, using a previously determined number from step one (*K* = 3). The program BayesAss 1.3 (Wilson & Rannala, 2003) was used to estimate rates of recent immigration among the Dhofar population. BayesAss assumes linkage‐equilibrium, and relaxes the assumption that the populations are in Hardy–Weinberg or migration‐drift equilibriums. We assumed contemporary gene flow to span the last five generations (i.e., 20–25 years) based on a generation time of 4–5 years (Dutta et al., 2013). The analysis was performed using five independent runs with different randomly generated starting seeds to ensure consistency between runs. We adjusted the delta values and used 50,000,000 iterations with a burn‐in of 5,000,000, sampling every 2000 iterations. To test the reliability of our data, we compared our migration rate estimates with the mean and 95% confidence intervals (CIs) expected for uninformative data that are provided by BayesAss.
- *Measuring temporal changes in genetic diversity and effective population size within the Jabal Samhan population*. To estimate temporal changes in genetic diversity, we use genotyped individuals from Jabal Samhan. This is the largest region where leopards are present and represent their last stronghold in Oman (Spalton & Willis, 1999). Consequently, it is presumed to support a greater number of individuals than Jabal Qamar, Jabal Qara, and the Nejd. To compute a time series of genetic diversity for Jabal Samhan, genotypes were separated by date into three temporal periods. Collection dates ranging between 1977 and 1985 were grouped to represent the '1985' period; 1997–2006 were grouped to represent the ‘2006’ period; 2012–2017 were grouped to represent the ‘2017’ period (Table S3). Hereafter, the 2017 period is synonymous with our use of the term “contemporary samples.” Data for the Jabal Samhan population comprised sample collection spanning 40 years, thereby providing an opportunity to explore effective population size (*N*_*e*_) over this period. We used a Bayesian method in the programme TMVP (Beaumont, 2003) which estimates a change in *N*_*e*_ across time. For full details, see Supplementary Information.

#### 2.5.3 Objective 3: Estimate density and size of the wild population

We applied a maximum‐likelihood SECR approach (Borchers & Efford, 2008), implemented in the R package SECR v3.1.3 to estimate leopard density in the Dhofar mountains using the molecular genotype data obtained from scat surveys, and individual photographic identification of leopards from camera trapping surveys. Two input files per data set were generated to run the analysis, comprising a capture history of individual leopards and their spatial detections. Spatial detections comprised (i) the coordinates of cameras, and (ii) the scat survey areas divided into 1 × 1 km grid cells with the center point of these cells used as a location. We estimated leopard density for Jabal Samhan (camera trap + scat) and Jabal Qamar (scat) only, due to sampling limitations for Jabal Qara and the Nejd (where the minimum sampling threshold for SECR of >10 captures was not met). Overall density derived from the scat data set was extrapolated to estimate leopard population size across the entirety of suitable habitat in the Dhofar mountains. We estimated leopard core habitat in Dhofar to be 2213 km^2^ and extrapolated the density estimate derived for Jabal Samhan and Jabal Qamar to produce an estimate for the full extent of habitat. Although leopards were recently recorded in the northern wadis of the Nejd, we consider this region marginal habitat, and it was excluded to avoid overestimating population size. For full details, see Supplementary Information.

#### 2.5.4 Objective 4: Assess the potential risks and benefits of genetic rescue for the wild population

We applied three distinct modeling approaches that complemented each other. First, simple computer simulations in Minitab 12.1 were used to examine the impact of releases on the allelic diversity. This approach is useful for conservation genetic studies that do not have access to whole‐genome sequence data. Here, we focused on allelic diversity rather than on heterozygosity or inbreeding coefficients because doing so made our data directly comparable to recent reintroduction studies (e.g., Jackson et al., 2022). In addition, simulations have shown that for populations with an ancestral *N*_*e*_ > 10,000, allelic diversity is a more sensitive metric (Hoban et al., 2021). Second, we used Vortex to simulate growth or decline of the wild population because it is the most commonly used software for population viability analysis (PVA) (Lacy & Pollak, 2023). Third, we complemented our Vortex analyses with computer simulations in SLiM (Haller & Messer, 2019). Like Vortex, this forward‐in‐time, individual‐based‐model enabled us to simulate the impacts of conservation action on population viability many generations into the future (Bertorelle et al., 2022; Femerling et al., 2023; Jackson et al., 2022). Using SLiM, we were also able to simulate the ancestral population size, rates of decline, and contemporary population sizes based on the known demographic history of the Arabian leopard (Al Hikmani et al., 2023). This is important because the past demography largely determines the size and composition of the genetic load (i.e., the masked load and realized load), as well as the distribution of selection and dominance coefficients present in the population (Dussex et al., 2023).

The impact of genetic rescue on the allelic richness of the wild recipient population was assessed through analysis of the effects of the number of released individuals (*n* = 1–6) and by using three different source populations: (1) Oman‐sourced captive leopards, (2) Yemen‐sourced captive leopards, and (3) total captive leopard population. We chose to focus here on the change in allelic richness because it is a measure that is more closely associated with a population's long‐term evolutionary potential. Furthermore, heterozygosity is not a sensitive metric for highly polymorphic microsatellite loci. Our simulation model tallied the number of unique alleles present at the eight microsatellite loci in the genetically rescued populations. Given the small number of microsatellite loci, the conclusions drawn from these simulations need to be interpreted with caution. Each rescue scenario was repeated 100 times, and the mean number of unique alleles and the 5%–95% CI were calculated. The model is available here as a macro in Minitab 12.1.

Simulations of growth or decline of the wild population following alternative scenarios of supplementation via reintroduction were performed using Vortex (version 10.6.0, Lacy & Pollak, 2023). Vortex was parameterized to include subpopulation allele frequency data from the captive and wild populations (see Objective 1), an initial wild population of 51 (95% CI: 32–79; see Objective 3) and life history parameters drawn from ecological studies of Arabian leopard (See Table S4). Vortex simulations were repeated to explore the sensitivity of parameters, including by varying mortality rate (10%–25%), and numbers of individuals from the captive population reintroduced to supplement the wild population (0 [no supplementation], 2, 6, and 10 individuals, every 5 years). We ran simulations with two values for the number of lethal equivalents (where one lethal equivalent corresponds to a group of deleterious alleles that would cause one death on average if made homozygous). The initial value was maintained at the Vortex default value of LE = 6.29. This value has been selected by the authors of the software to represent a reasonable estimate of the impact of inbreeding in wild populations. We then ran our simulations at double the default value (LE = 13.58) to reflect the accumulation of inbreeding depression likely in this population (Szulkin et al., 2007).

Simulations to assess the impact of a genetic rescue regime on neutral nucleotide diversity, genetic load, realized load (Bertorelle et al., 2022; Mathur & DeWoody, 2021), and fitness used Wright‐Fisher models in SLiM 3.0 (Haller & Messer, 2019). We aimed to determine the optimum number of Arabian leopards to be released from a captive population into a wild population. The simulated populations comprised diploid individuals, with an equal sex ratio of males and females. Their genomes consisted of an exome comprising nine pairs of autosomes, each containing 1000 genes of 1500 nucleotides. This chromosomal arrangement method has been employed in previous studies such as those conducted by Beichman et al. (2023) and Xie et al. (2022). Autosomal recombination was freely permitted, with a recombination rate of 1e‐3 between genes on shared chromosomes and 1e‐9 within genes. Deleterious mutations were introduced at a rate of 2.4e‐8, derived from a previously modeled stable population, with their distribution of DFE and dominance detailed in the Supplementary material. Neutral mutations were superimposed upon completion of the simulations at a rate of 1.6e‐8. Consequently, the ratio of deleterious to neutral mutations stood at 3:2. Population creation involved a two‐stage burn‐in process. The initial stage consisted of a neutral burn‐in, spanning 990,000 generations without deleterious mutations. This phase ensured the populations coalesced effectively, facilitating the correct overlaying of neutral mutations. Subsequently, the second stage of burn‐in, lasting 10,000 generations, aimed to establish a stable masked load of approximately 6 lethal equivalents. The ancestral population size, rates of decline, and contemporary population sizes were parameterized according to known demographic history of the Arabian leopard (Al Hikmani et al., 2023). We simulated the release of 0, 2, 4, 6, or 8 captive individuals (with a 1:1 sex ratio) every generation (5 years). For full details of SLiM simulations, see Supplementary Information. The script for the model is available here.

## 3 RESULTS

From 477 putative wild leopard scats, mtDNA identified 161 scats (34%) as Arabian leopard. The remaining 316 (66%) likely comprise caracal, Arabian wolf, and striped hyena. Our total sampling therefore numbered 214–161 scat samples, plus 53 from Arabian leopards of known provenance comprising of blood, skin, and scat (Tables S5 and S6).

### 3.1 Microsatellite amplification and identification of individuals

Of 35 markers applied to the DNA sample set, eight produced suitably scoreable genotypes and were observed to be polymorphic. Ten loci—of which seven are known to be polymorphic in other leopard subspecies (Mondol et al., 2009; Sugimoto et al., 2014; Uphyrkina et al., 2001)—were found to be monomorphic in Arabian leopard samples (Table S7). Remaining loci either did not amplify consistently (four loci) or failed to amplify (13 loci). No evidence of false, null alleles, or scoring errors were found, but we observed a small rate of dropout from scat samples: mean dropout rate 0.048 (range = 0.00–0.08).

Deviation from Hardy–Weinberg equilibrium was observed in marker 6HDZ700 in the Jabal Qamar population, marker 6HDZ89 in wild‐born samples originating from Yemen, and markers 6HDZ700 and FCA279 in the captive populations (derived from captive leopards from Oman, KSA, and UAE breeding centers). All loci were checked for null alleles using Micro‐Checker (Van Oosterhout et al., 2004), and none were found. No deviation from linkage disequilibrium was observed for any pair of loci.

Of 161 leopard scats, 109 were amplified for more than five loci, with a total of seven loci observed to be polymorphic in the wild population plus an additional locus polymorphic in the captive population. These eight loci were selected for further analyses (Table S7). To determine the power of seven polymorphic loci in the Dhofar population (FCA90, FCA105, FCA126, FCA279, 6HDZ89, 6HDZ635, 6HDZ700), genotypes of the 109 samples were assessed alongside those of leopards of known provenance and were used to identify any siblings present within the data set. The cumulative power of these loci to identify siblings improves as the number of loci increases, from PID (sib) 0.44 (one locus) to PID (sib) 0.02 (seven loci).

Five loci (FCA279, FCA105, 6HDZ700, 6HDZ635, 6HDZ89) produced genotypes that were distinguishable between individuals, including three siblings. The power of these loci in the scat data set was PID (sib) 0.05, which corresponds to one in every 20 leopards as a minimum for individual identification. We assumed, based on earlier camera trap work, that none of our four studied regions in Dhofar has more than 20 leopards (Spalton, Al Hikmani, Jahdhami, et al., 2006; Spalton, Al Hikmani, Willis, & Bait Said, 2006). We therefore used the program Cervus to identify the number of individual leopards within the scat genotype data set using a minimum of five loci. Following this approach, we identified the presence of 36 individual leopards among the 109 scat samples that amplified for more than five loci (Figure 2; Table S6): 10 following opportunistic collection (spanning 2012–2016), 20 through dedicated surveys (2017), and 6 arising from both collection modes. These 36 leopards comprised 17 from Jabal Samhan, 5 from Jabal Qara, 11 from Jabal Qamar, and 3 from the Nejd. The felid‐specific PCR primers designed to amplify the amelogenin regions of the y‐chromosome (Pilgrim et al., 2005) unambiguously identified sex for all of these individuals (23 males, 13 females).

### 3.2 Objective 1: Global patterns of genetic diversity

A summary of measures of global patterns of genetic diversity across the different populations is presented in Table 1.

- *Comparison of genetic diversity between the captive and wild population*. A total of 27 alleles were detected across the final data set, of which 17 alleles were shared between the wild population in Oman and the captive population (including those captive individuals wild‐born in Yemen). A total of eight alleles were unique to the captive population, of which two were unique to the Yemen‐sourced individuals. Two alleles were detected in the wild population that are not found in the genotyped individuals from the captive population. No unique alleles were found in museum samples (Table S1). The number of alleles per locus ranged from 2 to 5 with a mean of 3.38 alleles per locus (SE ± 0.38). The highest level of uHe and effective number of alleles was observed in the captive Arabian leopard population followed by the wild‐born captive leopards from Yemen (Table 1). These populations also have the highest allelic richness.
- *Comparison of genetic diversity between wild Oman and wild Yemen leopards*. The wild Oman population (across all four regions) had both the lowest genetic diversity (uHe ranges from 0.387 to 0.479) and lowest allelic richness (Ar ranges from 1.89 to 2.13). However, comparisons of these two genetic parameters (uHe and allelic richness) between Oman vs captive and Oman vs Yemen populations were not significant (Table 1). Similarly, no regional difference was observed between wild Omani leopards (Table 2; *p* = 0.85). Temporal genetic diversity and allelic richness were reduced in the Jabal Samhan population from uHe 0.438 in 1979 to uHe 0.387 in 2017 (Table 3) but not significantly so (*p* = 0.88). A single unique allele from a leopard wild‐caught in 1985 was not found in contemporary samples. AMOVA analysis of the population sets (wild Oman vs captive; wild Oman vs wild Yemen; the four Dhofar populations) indicated that the majority of overall variation is found within populations (Table 4).

**TABLE 1:** Measures of global patterns of genetic diversity in Arabian leopards. Samples are from the wild population (Oman), captive population (Oman, UAE, and Saudi Arabia) and captive leopards wild‐born in Yemen (this analysis is based on samples that genotyped for at least five loci).

Population	*N*	Na	*N*_*e*_	uHe	Ar
*Wild Oman*	45	2.375 ± 0324	1.952 ± 0.258	0.429 ± 0.076	2.35
*Captive (including 8 wild‐born in Yemen)*	33	3.125 ± 0.295	2.436 ± 0.243	0.571 ± 0.042	3.13
*Wild‐born Yemen (extracted from 33 captive samples)*	8	2.875 ± 0.227	2.202 ± 0.189	0.556 ± 0.052	2.88

*Note*: ANOVA test: wild Oman vs captive population *p* = 0.13/wild Oman vs wild Yemen, *p* = 0.19.

Abbreviations: Ar, allelic richness; *N*, sample size; Na, no. alleles; *N*_*e*_, no of effective alleles; uHe, unbiased expected heterozygosity.

**TABLE 2:** Contemporary levels of genetic diversity in different regions of Dhofar Governorate (This analysis is based on scats from 36 individual leopards collected between 2012 and 2017 from Dhofar, Oman).

Population	*N*	Na	*N*_*e*_	uHe	Ar
*Jabal Samhan*	17	2.250 ± 0313	1.791 ± 0257	0.387 ± 0.076	1.91
*Jabal Qara*	5	2.000 ± 0.189	1.795 ± 0.210	0.431 ± 0.038	1.89
*Jabal Qamar*	11	2.125 ± 0.227	1.762 ± 0.212	0.396 ± 0.074	1.89
*Nejd*	3	2.125 ± 0.227	1.789 ± 0.201	0.479 ± 0.089	2.13

*Note*: ANOVA test *p* = 0.85.

Abbreviations: Ar, allelic richness; *N*, sample size; Na, no. of alleles; *N*_*e*_, no. of effective alleles; uHe, unbiased expected heterozygosity.

**TABLE 3:** Time‐series of measures of genetic diversity in Jabal Samhan population.

Population	*N*	Na	*N*_*e*_	uHe	Ar
*Samhan 1985*	4	2.125 ± 0.227	1.792 ± 0.207	0.438 ± 0.087	2.13
*Samhan 2006*	5	2.125 ± 0.227	1.768 ± 0.181	0.433 ± 0.074	2.09
*Samhan 2017*	17	2.250 ± 0.313	1.791 ± 0.257	0.387 ± 0.076	2.05

*Note*: ANOVA test *p* = 0.88. Abbreviations: Ar, Allelic richness; N, Sample size; Na, No. Alleles; *N*_*e*_, No of Effective alleles; uHe, Unbiased Expected Heterozygosity.

**TABLE 4:** Analysis of molecular variance (AMOVA) result between wild and captive populations of Arabian leopards.

Source	*df*	SS	MS	Est. Var.	%	*p*
Wild Oman vs captive population						
Among Pops	1	64.895	64.895	1.575	24	0.001
Within Pops	76	374.733	4.931	4.931	76	0.001
Total	77	439.628		6.506	100	
Wild Oman vs wild Yemen						
Among Pops	1	28.391	28.391	1.748	27	0.001
Within Pops	51	236.817	4.643	4.643	73	0.001
Total	52	265.208		6.392	100	
Different regions of Dhofar (Jabal Samhan, Jabal Qara, Jabal Qamar, Nejd)						
Among Pops	3	28.621	9.540	0.664	13	0.013
Within Pops	32	137.657	4.302	4.302	87	0.013
Total	35	166.278		4.966	100	

### 3.3 Objective 2: Spatio‐temporal patterns of genetic diversity

**FIGURE 3:**
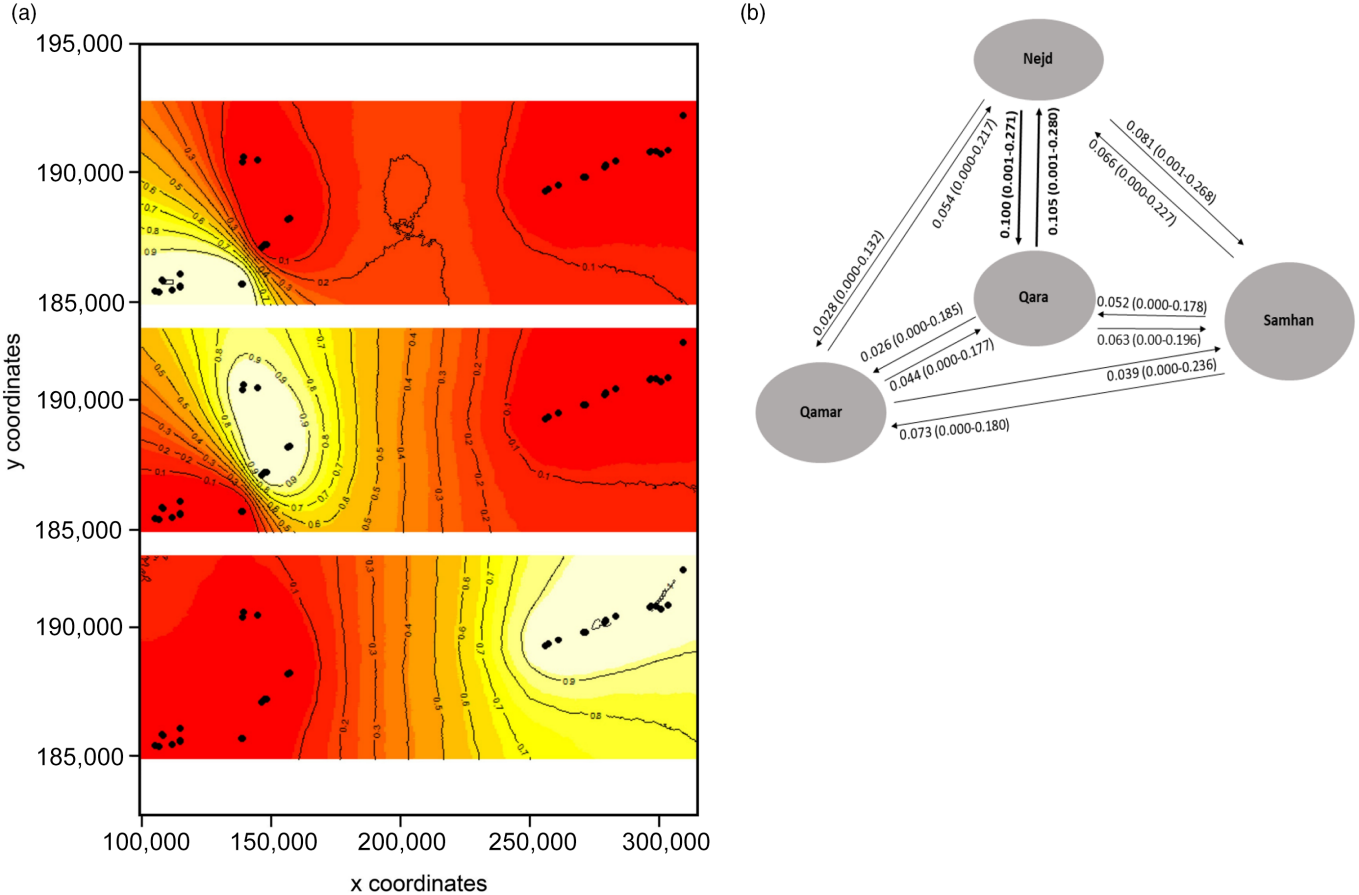
(a) Maps showing GENELAND individual assignments for 36 individuals typed at seven microsatellite loci. Membership values are in yellow and the level curves illustrate the spatial changes in assignment values. X/Y coordinates refer to latitude/longitude. (b) Patterns of contemporary gene flow in the wild population of Arabian leopards across the Dhofar region based on migration rates estimated using BayesAss (Wilson & Rannala, 2003). Arrows indicate the direction of migration and numbers above rows indicate migrations rates. Numbers in brackets indicate 95% confidence intervals. Bold numbers indicate significant gene flow.

**FIGURE 4:**
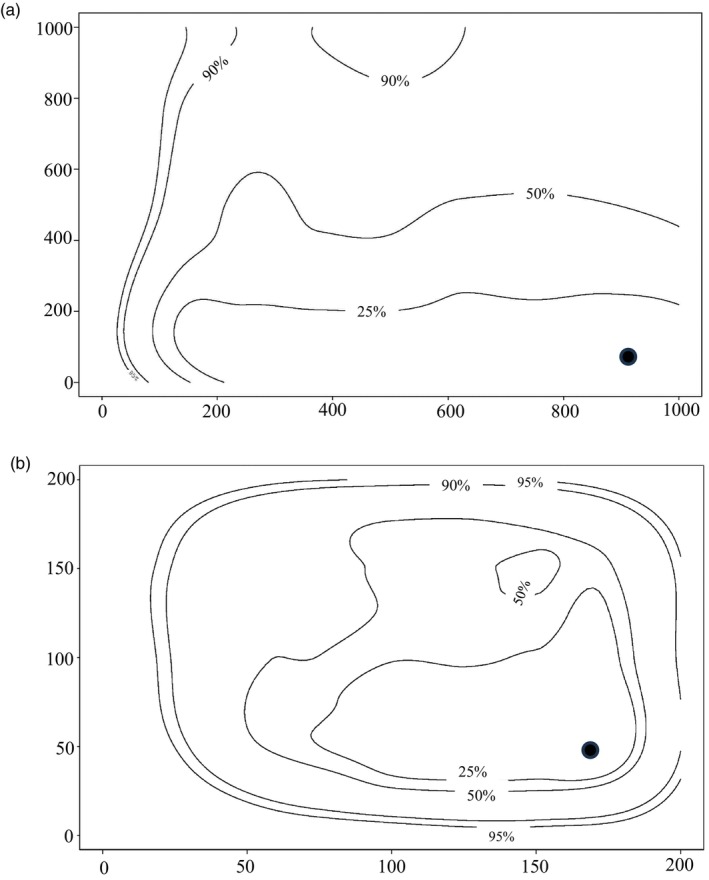
TMVP estimates of historical and contemporary effective population sizes (*N*_*e*_) for Jabal Samhan population following the methods of Beaumont (2003). (a) A rectangular prior of 0–1000, (b) refined rectangular priors of 0–200. The single black circle indicates the joint mode; contours indicate density limit of posterior distribution 25%–95%.

- *Measuring the extent of genetic differentiation between different regions within Oman*. Leopards from Jabal Qara, Jabal Qamar, and Jabal Samhan showed a significant level of population differentiation (the Nejd was excluded from the analysis due to its low sample size: *n* = 3). The greatest differentiation was observed between Jabal Qara and Jabal Qamar (*F*_ST_ = 0.108), followed by Jabal Samhan and Jabal Qamar (*F*_ST_ = 0.094), and Jabal Samhan and Jabal Qara (*F*_ST_ = 0.070). A Mantel test to assess the potential for isolation by distance (IBD) indicated significant correlation between genetic and geographic distance (*r* = 0.13964, P (rxy‐r and ≥rxy data) = 0.001; Figure S5a). A spatial autocorrelation analysis using GenAlEx showed significant positive autocorrelation when samples are geographically proximal, and significant negative autocorrelation when samples are more distant (see Figure S5b).
  Spatial analysis of genetic structure using GENELAND indicated the presence of three genetic clusters within Dhofar (Figure 3). Cluster 1 corresponds to Jabal Qamar, cluster 2 to Jabal Qara and the Nejd, while cluster 3 corresponds to Jabal Samhan. The probability of Jabal Qamar leopards belonging to cluster 1 was 94%–99%, while Jabal Qara and the Nejd had a probabilitiy of 94%–99% belonging to cluster 2. The leopards of Jabal Samhan had a 97%–100% probability belonging to cluster 3. Estimates of contemporary rates of migration (number of migrants per generation, Nm) between Dhofar populations, estimated using BayesAss, were generally low (mean Nm of 0.026–0.081; Figure 3b). Significant estimates of migration (Nm > 0.1) were detected from Jabal Qara to the Nejd and vice versa (0.100 and 0.105). The lowest levels of migration were from Jabal Qara to Jabal Qamar (0.026), and from the Nejd to Jabal Qamar (0.028).
- *Measuring temporal changes in genetic diversity and effective population size within the Jabal Samhan population*. Analysis using TMVP showed a proportional reduction of 90% from *N*_*e*_ = 853 for the historical population (95% limits 74–1000) to *N*_*e*_ = 81 for the contemporary population (95% limits 0–940; priors =0–1000), and a reduction of 67% from *N*_*e*_ = 168 for the historical population (95% limits 26–197) to *N*_*e*_ = 55 for the contemporary population (95% limits 12–192) when the model parameters were refined (priors = 0–200) to more closely reflect known biological reality (Figure 4). However, the 95% higher posterior limits (HPD) surrounding the proportional reductions indicate that an overall interpretation of reduction in *N*_*e*_ should be treated with caution.

### 3.4 Objective 3: Density and size of the wild population

From scat samples collected between January and April 2017, we identified 26 individual leopards, comprising 14 males and 12 females: Jabal Samhan (8:5), Jabal Qara (1:3), Jabal Qamar (4:3), the Nejd (1:1). Camera traps in Jabal Samhan accumulated 2665 trap days and recorded leopards at 21 out of 41 stations (one camera lost). A total of 397 photographs captured the presence of leopards (Table 5; Figures S4 and S6), of which 306 (77%) were suitable for individual identification. From these images 11 individual leopards (7:4) were identified. The remaining photographs (23%) were triggered at night and light levels rendered them unusable for purposes of individual identification. A total of 60 photographs of leopards were obtained from Jabal Qara and 85 from Jabal Qamar cameras. From these, we identified five individuals (3:2) in Jabal Qara and six individuals (3:3) in Jabal Qamar. Leopards were recorded in 5 of the 13 camera trap stations in Jabal Qara and in 12 of the 20 camera trap stations in Jabal Qamar. Numbers of individual leopards identified in each of the different regions were comparable between genetic‐ and camera‐derived methods (χ^2^ = 0.0069, *p* = 0.99) (Figure S7).

**TABLE 5:** Summary of Arabian leopard photographs/scat samples obtained from each of the sampling regions in Dhofar. Numbers in parentheses indicate the number of leopard photographs/scat samples that were used for individual identification.

Region	Method	Sampling duration (days)	No. of leopard photographs/scats	No. of individuals detected
Jabal Samhan	Camera traps	65 days	397 photographs (305)	11
	Scat sampling	35 days	76 scats (46)	13
Jabal Qara	Camera traps	84 days	60 photographs (42)	5
	Scat sampling	25 days	9 scats (8)	4
Jabal Qamar	Camera trap	100 days	85 photographs (74)	6
	Scat sampling	28 days	18 scats (13)	7
Nejd	Scat sampling	24 days	3 scats (2)	2

*Note*: Chi‐square test: number of leopards from scats vs camera traps (χ^2^ = 0.0069; *p* = 0.99).

Spatially explicit capture–recapture provided support for more than one model when data were analyzed independently per region (Table S8). When genetic data were combined across all regions to estimate overall density, the ‘constant detection probability and spatial movement’ model had strong support (Table S8). Based on the models ranked highest using AIC, analysis of the genetic data using SECR yielded an overall leopard density estimate of 2.30 ± 0.53 S.E leopard/100 km^2^ (Table 6), with the highest density of leopards estimated in Jabal Qamar (3.60 individuals ±1.57 S.E) followed by Jabal Samhan (2.03 individuals ±0.58 S.E).

**TABLE 6:** Arabian leopard density parameters estimates with spatially explicit capture–recapture (SECR) based on top‐ranked models.

Area‐method	Sex	Number of individuals detected	Effective sampling area (km^2^)	Density per 100 km^2^/(S.E)	Probability of detection/(S. E)	Spatial distance moved (S. E)
Jabal Samhan (camera)	F	4	733	2.65 (1.07)	0.052 (0.019)	7.56 (1.65)
	M	7			0.011 (0.006)	7.61 (1.80)
Jabal Samhan (scat)	F	5	733	2.03 (0.58)	0.059 (0.015)	8.64 (1.46)
	M	8			0.056 (0.013)	8.56 (1.42)
Jabal Qamar (scat)	F	3	298	3.60 (1.57)	0.077 (0.049)	2.85 (0.83)
	M	4			0.097 (0.064)	2.99 (0.91)
Overall based on the scat data set (Jabal Samhan and Jabal Qamar)		20	1031	2.30 (0.53)	0.050 (0.010)	7.98 (1.14)

Analysis of Jabal Samhan camera trap data estimated a higher number of leopards (2.65 ± 1.07 S.E leopard/100km^2^) than the genetic data. For Jabal Samhan detection probability for camera trap surveys was higher for females (0.052 ± 0.019 S.E) than males (0.011 ± 0.006 S.E). However, both sexes showed similar detection probability from genetic analysis (males: 0.056 ± 0.013 S.E; females 0.059 ± 0.015 S.E; Table 6). For Jabal Qamar, males (0.097 ± 0.064) had a slightly higher detection probability than females (0.077 ± 0.049). There was little variation between males and females in terms of their spatial movement within populations, but significant variation was observed between populations. Higher spatial movement estimates were recorded in the Jabal Samhan population (7.56–8.64 km) compared to Jabal Qamar (2.85–2.99 km).

Using an overall genetic density estimate of 2.30 ± 0.53 S.E leopard/100 km^2^, we calculated that the Dhofar mountains could currently be supporting 51 leopards (95% CI: 32–79), compared with 45 leopards (95% CI: 27–83) when applying the camera data to estimate overall density in Dhofar (2.06 ± S.E 0.07 leopard/100 km^2^).

### 3.5 Objective 4: Assess the potential risks and benefits of genetic rescue for the wild population

**FIGURE 5:**
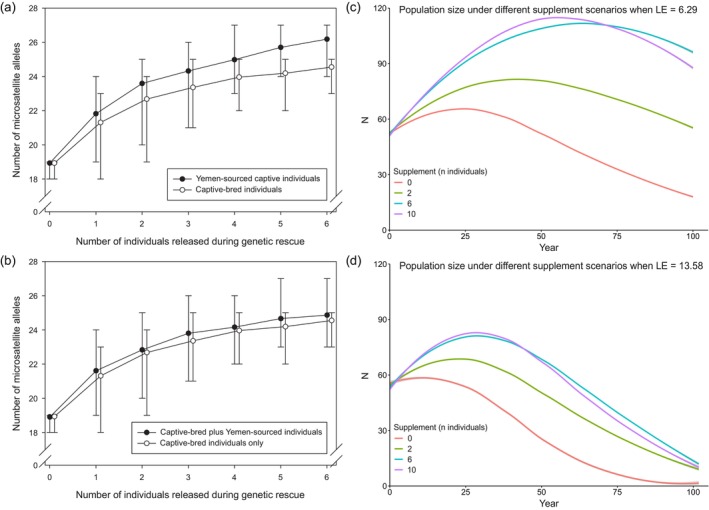
(a) Number of unique microsatellite alleles as a function of the number of individuals that are released during genetic rescue originating from the captive‐bred individuals only (open symbols) and from the captive‐bred plus the Yemen‐sourced captive population (solid symbols). (b) Number of unique microsatellite alleles as a function of the number of individuals that are released during genetic rescue originating from the Yemen‐sourced captive population (solid symbols) and the captive‐bred individuals only (open symbols). Vortex simulations were performed for the following scenarios: no supplementation (red), 2 individuals reintroduced every 5 years (green), 6 individuals/5 years (blue), 10 individuals/5 years (purple); parameterized specifying 20% mortality for 1–2 years, *k* = 500, and varying Lethal Equivalents, (c) LE = 6.29 and (d) LE = 13.58.

The release of a relatively small number of captive individuals has the potential to increase genetic diversity in the wild population (Figure 5a,b). The mean number of unique microsatellite alleles is predicted to increase from 19.0 to 24.6 by the release of six captive‐bred Oman‐sourced individuals, an increase of 29% (Figure 5a). If, in addition to Oman captive‐bred individuals, the Yemen‐sourced captive population was included during genetic rescue, genetic diversity would increase further, to a mean of 24.9 alleles (31% increase in diversity (Figure 5b)). If individuals from a purely Yemen‐sourced lineage were to be used exclusively as the source population, based on the proportion of novel alleles among those sampled, genetic diversity would increase yet further, to a mean of 26.2 alleles (38% increase in diversity; Figure 5a).

**FIGURE 6:**
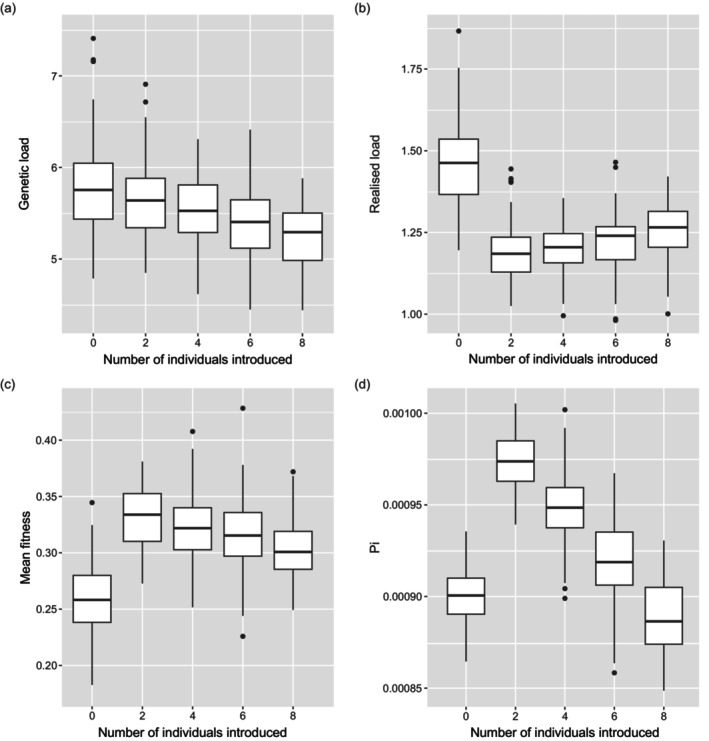
Boxplots showing the distribution of (a) genetic load (in lethal equivalents), (b) realised load (in lethal equivalents), (c) mean fitness, and (d) neutral nucleotide diversity (Pi) in a wild population after 20 generations, with the release of 0, 2, 4, 6, or 8 individuals from a captive population per generation (i.e., every 5 years). Shown are the medians, the interquartile range (boxes), the range (whiskers), and the outliers.

The Vortex simulations suggest that supplementation using the captive population is likely to improve the long‐term viability of the wild population, although there is an optimum size of annual release cohort above which population viability decreases. Long‐term viability appears to be strongly dependent on the genetic load. The simulated supplemented populations never fell below the starting population size when assuming LE = 6.29 (Figure 6c), whereas all scenarios showed a steep decline under an assumption of LE = 13.58 (Figure 6d).

Figure 6 illustrates the results from SLiM simulations 20 generations (i.e., 100 years) after release, showing the impact of genetic rescue on the genetic load, realized load, fitness, and nucleotide diversity of the wild population. The captive and wild population have diverged from one another due to reproductive isolation and drift. As a consequence of its small breeding population size (*N* = 64), some of the genetic load in the captive population has been purged. Consequently, releasing captive‐bred individuals reduces the genetic load in the rescued wild population almost linearly (Figure 6). However, releasing a relatively large number of captive‐bred individuals comes with a noticeable cost in terms of a loss in nucleotide diversity and increased realized load.

Indeed, the impact of reintroduction on the realized load shows a nonlinear relationship. This component of the load is minimized when two individuals are released every 5 years. If more than two individuals are released every 5 years, it ultimately increases the level of inbreeding in the rescued wild population (i.e., after 20 generations or 100 after the start of the release program). This increase in realized load is not immediately apparent, given that most matings are likely to occur between captive and wild individuals in the early generations after release. However, after 20 generations, the descendants of these released individuals are relatively more related, and that causes an increase in the realized load. This pattern is echoed by the effect on fitness and genetic diversity. Both these metrics are also maximized when two captive‐bred individuals are released every 5 years, and a larger number of releases is likely to accelerate genomic erosion of the wild population. Based on these simulations, we can conclude there is likely an optimum number of captive releases, and further analysis of the genetic load and genome‐wide diversity are required to determine the optimum conservation management plan.

## 4 DISCUSSION

We provide the first temporal and spatial assessment of inter‐ and intraspecific genetic diversity across wild and captive populations of the Critically Endangered Arabian leopard, alongside the first robust estimate of population size for Arabian leopards in Oman. Based on a small panel of microsatellite loci, we show that 29% of genetic diversity present in captive leopards is undetected in the wild population. Our simulations indicate that genetic rescue has the potential to increase the number of alleles in wild leopards and reduce the realized load and genetic load, thereby enhancing their long‐term population viability. However, there is likely to be an optimum number of captive releases, and exceeding this number could accelerate genomic erosion of the rescued wild population. We discuss the key findings in more detail below, mindful of the need for caution when interpreting the genetic results given the limited set of markers used in this study.

### 4.1 Objective 1 global patterns of genetic diversity

- *Comparisons of genetic diversity between populations of captive and wild individuals*. The eight polymorphic loci detected a total of 27 alleles of which eight were unique to the captive population including the wild‐sourced captive individuals from Yemen. Although not significant, the captive and Yemen populations also have greater genetic diversity than the Dhofar population of Oman which is considered the last stronghold for the Arabian leopard (Breitenmoser et al., 2010). The low genetic variation in the wild Arabian leopard population could be due to a recent population crash in combination with genetic drift during its historic population size decline. Prior to the 19th Century, the leopard was widespread across the western and central regions of the Arabian Peninsula (Harrison & Bates, 1991), with an undoubtedly larger population size (Figure 1). However, in the late 20th century, following the introduction of modern lightweight firearms and their use by herders, more than 100 leopards were reported killed (e.g. see Al Jumaily et al., 2006; Qarqz & Baker, 2006; Spalton, Al Hikmani, Jahdhami, et al., 2006; Zafar‐ul Islam et al., 2018), although this figure is probably a significant underestimation. Ultimately, targeted killing of leopards led to a decline in the population, and their extirpation from northern Oman in 1976 (Spalton, Al Hikmani, Jahdhami, et al., 2006), Jordan in 1987 (Qarqz & Baker, 2006) and the UAE in 2001 (Edmonds et al., 2006). It is therefore reasonable to posit that the Arabian leopard has been subject to a prolonged genetic bottleneck (Mochales‐Riaño et al., 2023), and that this likely resulted from a human‐mediated population crash, which would explain the loss of genetic diversity.
- *Comparisons of genetic diversity between wild Oman and wild Yemen leopards*. Despite the small sample size of leopards from Yemen, the captive population contained 8 unique alleles that are not found in the Oman wild population, likely a result of isolation and restricted gene flow (Allendorf et al., 2013) as the once contiguous leopard population of south Arabia has become increasingly fragmented (Breitenmoser et al., 2006). We believe that some of the Yemeni samples were from leopards captured from the Wada'a region in northwest Yemen (Figure S8) that were taken to Yemeni zoos and later to other regional collections (Al Jumaily et al., 2006). Wada'a is at least 900 km from Dhofar, and although in the early 20th Century there may have been some connectivity of leopard habitat to enable dispersal and gene flow between southern Oman and northern Yemen, today this is unlikely due to habitat fragmentation (Figure 1).

In the absence of dispersal, region‐specific genetic identity can develop in isolated populations. However, for substantial variation to occur in an isolated population, it not only requires isolation for many generations, but the population must persist at a sufficient effective size to retain those alleles. Populations in southern Oman and northern Yemen are believed to have been isolated for a considerable period but the Yemeni population either remained large enough to retain these unique alleles, generated them through de novo mutations or had new alleles reintroduced through gene flow and connectivity with other leopard populations (such as the Sarawat mountains in Saudi Arabia). If the Yemeni population persists, further sampling from this population may help to clarify whether more alleles detected in Yemeni leopards are unique or shared with leopards found in Saudi Arabia. If unique alleles came from a larger population in Yemen, it will be important to know its current geographic range and extent of occurrence and to establish how leopards have managed to survive in light of their extirpation from elsewhere in the region. Yemen has a considerable human population (around 33 million; World Bank, 2021) but over the last 100 years, largely because of limited economic development and several extended conflicts, much of the country has remained remote and undeveloped in comparison with other countries of the region. Under these conditions, the Arabian leopard may have persisted in greater numbers in remote mountainous areas. Currently, there are no reliable data on the status of wild leopards in Yemen, but sightings and killing of leopards have been reported from several areas across the country in recent years, in particular from the south and east (Al Hikmani & Spalton, 2023). Knowledge of the number of Yemeni leopards, their geographic extent, and levels of genetic diversity will be invaluable for future conservation efforts.

### 4.2 Objective 2: Spatiotemporal patterns of genetic diversity

- *Measuring the extent of genetic differentiation between different regions within Oman*. Despite the low number of loci found to be polymorphic, our marker set revealed detectable spatial genetic structure within the leopard population of Dhofar, providing important insight into how habitat fragmentation and isolation have led to genetic differentiation. GENELAND grouped leopards from the Nejd with Jabal Qara and identified Jabal Samhan as a third cluster. The extent of differentiation appears most pronounced between the leopards of Jabal Qara and Jabal Qamar where we also detected low levels of migration (Figure S9).
  The pronounced genetic differentiation observed between leopards of Jabal Qara and Jabal Qamar is supported by the GENELAND analyses. Jabal Qara and Jabal Qamar are geographically proximal, and the leopards of these two regions would be expected to show reduced differentiation unless there exists some barrier between them. We are unaware of any substantial biogeographic barrier between Jabal Qara and Jabal Qamar and instead consider anthropogenic disturbance, including settlements and large numbers of livestock, to restrict leopard movement. Three further aspects of human activity may explain this intriguing result of relatively localized differentiation. First, roads are known to limit carnivore movement and cause population subdivision (Forman & Deblinger, 2010; Lesbarrères et al., 2006; Ngoprasert et al., 2007; Riley et al., 2006). It is possible that the Salalah‐Sarfayt road that runs through Jabar Qamar, in combination with associated settlements and livestock herds, has restricted leopard movement and induced genetic differentiation between Jabal Qamar and Jabal Qara populations (Figure S9). Second, the Dhofar conflict between 1965 and 1975 (Hughes, 2009), including the construction of the 50 km “Hornbeam” defence line in 1973, at the western end of Jabal Qara may have played a role in isolating leopard populations (Figure S10; Tusa, 1988). Built of barbed‐wire and landmines, the “Hornbeam line” was designed to prevent rebels crossing from Jabal Qamar to Jabal Qara for incursions on the town of Salalah. Although the defence line was dismantled in the late 1970s, it may have restricted leopard movements, preventing dispersal and resulting in genetic differentiation. Third, across the Dhofar region, rapid development and huge increases in livestock numbers are considered to have had negative impacts on this landscape (Ghazanfar, 1998; Miller & Morris, 1988). Given that large carnivores are sensitive to human development and disturbance (Smith et al., 2015; Woodroffe, 2000), such impacts likely explain some of the current patterns of Arabian leopard distribution. Prior to 1970, the level of human impact on the Dhofar environment was considered low (Shaw Reade et al., 1980), but it has since seen rapid development, and human settlements have been built throughout Jabal Qara, on the northern plateau and coast of Jabal Qamar, and along the foothills of Jabal Samhan.
  The only significant indication of connectivity that our study revealed was between western Jabal Qara and the Nejd. This result is unsurprising as some of the leopards that have been recorded in the Nejd were recently photographed in western Jabal Qara (Office for Conservation of the Environment, unpublished data; Figure S11). In contrast to the eastern and central parts of Jabal Qara, which are separated from the Nejd by 20–30 km of monsoon rangeland heavily used by people and their livestock, the mountains in western Jabal Qara are narrow and the distance to the dry north wadis of the Nejd is short (~5 km). In these conditions, leopard movement and gene flow between the Nejd and western Jabal Qara is conceivable (for details of management recommendations to facilitate habitat connectivity, see Supplementary Information).
- *Measuring temporal changes in genetic diversity and effective population size within the Jabal Samhan population*. The observed (nonsignificant) temporal loss of genetic diversity in Jabal Samhan across the 40‐year time period (11.6% reduction in uHe from 43.8% to 38.7%, and an observed loss of a single allele) is reflected in the observed temporal decline in *N*_*e*_ (a 67% reduction, from *N*_*e*_ = 168 to *N*_*e*_ = 55). While the observed loss of diversity over time is not significant, it may be nontrivial in that further loci might confirm this loss as substantial. Indeed, the observed decline in uHe equates to an increase in mean inbreeding coefficient F by 0.116 (from what was probably an inbred starting point) which is within the range where inbreeding depression is expected (Frankham et al., 2017). Recent conservation measures, such as the banning of leopard killing since 1976, establishment of the Jabal Samhan Nature Reserve in 1997, and public awareness programs and compensation schemes for livestock herders to reduce leopard killing, may have slowed the rate of population decline and loss of genetic diversity (Al Hikmani, 2018), however, our findings align with expectations for a small, fragmented population of a Critically Endangered species. While the number and nature of the loci used in this study require these findings to be interpreted with a degree of caution, conservation management decisions often need to be made with imperfect data.

### 4.3 Objective 3: Density and size of the leopard population

#### 4.3.1 Estimation of leopard density

Our findings from Jabal Samhan show that noninvasive DNA sampling can provide estimates of density that are comparable with estimates derived from camera trap data. We found that both survey techniques identified broadly comparable numbers of individual leopards in each mountain region (Table 4 and Figure S7). However, camera traps overestimated density in Jabal Samhan in comparison with genetic sampling despite both techniques recording a similar number of leopards (camera traps = 11; genetic sampling = 13). Yet, the estimate from genetic sampling in this region has higher precision as the coefficient of variation (CV; SE/density) was lower (28%) in comparison with that from camera traps (40%). The variation in density estimates between the two methods may have been due to the lower number of leopard detections/captures obtained from camera traps (camera trap = 27 detections; genetic sampling = 46 detections; Figure S6). Our scat surveys are likely to have covered more spatial ground in terms of their ability to record leopard presence beyond the fixed locations of camera traps, and in doing so, the scat sampling approach obtained more data than camera traps, which only record passing animals. Therefore, our density estimates from the genetic sampling are probably more representative of the true density of leopards in Jabal Samhan.

The overall density estimate of 2.30 leopards/100 km^2^ for the Dhofar mountains is comparable to estimates for other threatened leopard populations. Vitkalova et al. (2018) reported a density estimate of 1.4 leopards/100 km^2^ for the Critically Endangered Amur leopard *Panthera pardus orientalis* in Russia and China, whereas Thapa et al. (2014) reported estimates of 3.78 leopards/100 km^2^ for the Indian leopard *Panthera pardus fusca* in Nepal. The low‐density estimate for the Arabian leopard is likely to be an indication of their low numbers (Jacobson et al., 2016).

Genetic sampling provided more precise estimates of detection probability and spatial movement than the camera trapping method in Jabal Samhan. Yet, both techniques show that male and female leopards have similar spatial movement patterns in Jabal Samhan, and scatology found this to also hold true for Jabal Qamar. However, the leopards of Jabal Samhan exhibit larger spatial movement patterns than the leopards of Jabal Qamar, indicating interpopulation variation. Home range and movement patterns are often larger for males than for females in most territorial carnivores, but Marker and Dickman (2005) did not find significant difference in range size between male and female leopards in Namibian farmlands. The only previous estimate of home range for the Arabian leopard is derived from a study in which GPS collars were fitted to two individuals, a male from Jabal Samhan and a female from Jabal Qamar (Spalton & Al Hikmani, 2014). This study estimated home range to be 168 km^2^ for male leopards and 64 km^2^ for female leopards, with average daily movement to be 8.5 and 3 km, respectively. Our study did not find evidence for difference in spatial movement between male and female leopards, but we did detect differences between populations, with high levels of spatial movement within Jabal Samhan. If this interpopulation variation is a true reflection of differences in spatial movement then it may be explained by differences in habitat. Jabal Samhan comprises semi‐ to hyper‐arid habitat and leopards of this region may need to travel large distances to find food and water in comparison to Jabal Qamar. Jabal Qamar is located in the monsoon zone, and though both Nubian ibex and Arabian gazelle are absent, the greater primary productivity of the monsoon forests is likely to support greater numbers of small prey such as the rock hyrax.

Our study indicates that Jabal Qamar harbors a higher leopard density than Jabal Samhan. The population density of any large carnivore is associated with the density of its preferred prey species (Hayward et al., 2007; Karanth et al., 2004), and this relationship is in turn associated with rainfall and vegetation productivity (East, 1984). Although no large ungulates are found in the woody slopes and plateau grasslands of Jabal Qamar (Spalton & Al Hikmani, 2014), several small prey species such as rock hyrax, porcupine, and rodents are found within this region (Spalton & Al Hikmani, 2014), and though we do not know the density of these known prey species, they are likely abundant (HAH. pers. obs). In addition, livestock including camels and cattle, are present in substantial numbers in Jabal Qamar and are known to provide an alternative food source for the leopard; several cases of leopard livestock predation have been confirmed by camera traps in this locality. A diet of plentiful small prey species supplemented with livestock may allow for a higher leopard density in Jabal Qamar than in the elevated arid region of Jabal Samhan.

### 4.4 Estimation of leopard population size

Our estimate of 51 leopards (95% CI: 32–79) in the Dhofar mountains aligns with a previous estimate in 2014 of 44–58 leopards in Dhofar. The previous estimate was based on the first camera trap surveys for this species comprising data from 1997 to 2000 and GPS collar data from leopards caught between January and March 2002, December 2003 and February 2004, and July and August 2005 (Spalton & Al Hikmani, 2014; Spalton, Al Hikmani, Jahdhami, et al., 2006; Spalton, Al Hikmani, Willis, & Bait Said, 2006). Similarly, the 26 leopards we identified from scats are comparable to the number reported by Spalton, Al Hikmani, Jahdhami, et al. (2006) and Spalton, Al Hikmani, Willis, and Bait Said (2006) in the same region (Jabal Samhan: *N* = 17; Jabal Qara‐Qamar: *N* = 9–11). If these results are a true reflection of population size, they suggest that the Dhofar population may have remained somewhat stable for the last two decades, but they confirm that the population size remains perilously small and highly vulnerable to increasing anthropogenic pressure on these leopards and their habitat. The small effective population size is likely to result in continued genomic erosion and a loss of diversity due to a drift debt (Pinto et al., 2024). Such drift debt is typical for gene pools that have not yet reached their mutation‐drift equilibrium, and it could further jeopardise the long‐term viability of this population (Pinto et al., 2024).

### 4.5 Objective 4: Potential for genetic rescue using captive stock

Genetic rescue has been successful for several threatened taxa including the Florida panther, Mexican wolves, and Swedish adders, increasing levels of genetic diversity and fitness within their natural populations (Hedrick & Fredrickson, 2010; Johnson et al., 2010; Madsen et al., 2020). The importance of conservation breeding and reintroduction of the Arabian leopard has been highlighted in the *Strategy for the Conservation of the Leopard in the Arabian Peninsula* (Breitenmoser et al., 2010), with the aim to produce a viable and sustainably managed population of Arabian leopard. The captive population is crucial in achieving this aim (Breitenmoser et al., 2006).

Our simulations indicate that supplementation of the wild population by reintroduction of individuals from the captive population can substantially increase the mean number of microsatellite alleles in the wild population by between 29% and 38%, returning variation that no longer exists in the wild. Although microsatellites are noncoding markers they are highly polymorphic, which makes them very suitable for detecting changes in population demography (Barson et al., 2009), and for assessing the impact of genetic rescue (Miller et al., 2020). The number of unique alleles is a particularly sensitive population genetic summary statistic, more so than, for example, heterozygosity or nucleotide diversity, which tends to respond at a much slower rate to changes in effective population size. Although microsatellite alleles are considered to be neutral, they are broadly representative of genetic variation that could be adaptive and contribute to the evolutionary potential of the population in response to environmental change. Furthermore, microsatellite variation is also positively correlated to immunogenetic diversity in some natural systems (Santonastaco et al., 2017), although the changes in variation at immune genes of the MHC can proceed even more rapidly than those at microsatellite loci (Eimes et al., 2011). The loss of alleles during population size decline can be considered “mini‐extinctions”, but such genomic erosion can be offset by genetic rescue, supplementing the population with new variation. However, on a more cautionary note, these conclusions are based on a small panel of eight microsatellite loci, and they should be expanded on with estimates of genome‐wide diversity.

Our Vortex simulations suggest that supplementation using the captive population is likely to improve the long‐term viability of wild leopards, but above an annual release cohort of six individuals every 5 years, population viability decreases. This is possibly due to relatedness among the captive‐bred individuals which will result in a higher realized load of their offspring. More importantly, long‐term viability appears strongly dependent on the genetic load (Figure 6). Together, our simulations highlight the potential of a genetic rescue effect by using the captive population, but also a need for whole genome analyses to thoroughly understand the evolutionary genomic consequences of the different scenarios for genetic rescue. Increasingly, calls are made for evidence‐based risk assessments for species that could benefit from genetic rescue, but which may have been reproductively isolated (e.g., see Krojerová‐Prokešová et al., 2023; Parmesan et al., 2023; Pavlova et al., 2023).

Genetic rescue may help to mask some of the genetic load and increase fitness (Bertorelle et al., 2022), and can provide the rescued population with new variation to adaptively respond (i.e., evolutionary rescue). However, there are several risks. First, there is a risk of genetic or genomic incompatibilities between Yemen and Oman populations that could have evolved during reproductive isolation. Indeed, initial divergence dating indicates that leopards from Yemen and Oman comprise two distinct lineages that diverged ~147 kya (65–243 kya) (Al Hikmani, 2019), a duration of independent evolutionary history that may result in genomic incompatibilities following introgression. Second, the unintended introduction of deleterious mutations unique to captive‐bred individuals could increase the genetic load. This risk may be (partly) reduced by the apparent purging of deleterious alleles in the Arabian leopard which probably resulted from increased inbreeding (Mochales‐Riaño et al., 2023). However, although severely deleterious mutations (lethal and semi‐lethal variants) are likely to be purged by inbreeding in captivity, moderate effect mutations might increase in frequency due to the relaxed purifying selection in the small captive population. Our simulations assumed a default value of a genetic load of 6.29 LEs, which is an average across wild mammal and bird populations (O'Grady et al., 2006). However, the estimates vary widely between species. We therefore also ran simulations doubling this number, which is similar to the estimated genetic load for the pink pigeon (Jackson et al., 2022). These simulations highlighted the critical effect of the genetic load on the long‐term viability of the leopard, which illustrates the importance of estimating and accounting for genetic load in genetic rescue. A conservation management program would therefore greatly benefit from estimating the genetic load and incorporating these data into computer simulations to assess different genetic rescue scenarios (Bertorelle et al., 2022; Speak et al., 2023; Van Oosterhout, 2020). We furthermore acknowledge that it will be important to empirically validate the impact of any genomics‐informed recommendations by intensely monitoring the populations of the Arabian leopard during and after genetic rescue.

Our SLiM simulations illustrate the benefits of genetic rescue and its potential to reduce the genetic load (Figure 6a) and the realised load (Figure 6b), whilst also improving mean fitness (Figure 6c) and nucleotide diversity (Figure 6d) in the wild population. However, for some genomic metrics, there is an optimum number of released individuals. Reintroducing two individuals is optimal for reducing realised load and maximizing fitness and nucleotide diversity. Reintroducing more captive‐bred individuals appears to be suboptimal. This is because the captive population and wild population possess deleterious mutations at different loci. When a relatively small number of captive individuals are released, this optimizes the masking of deleterious alleles at different loci in the rescued wild population. Exceeding this optimal number increases homozygosity of deleterious mutations that are unique to the captive population. Similarly, neutral nucleotide diversity (Pi) is maximized when just two captive individuals are released every generation (i.e., every 5 years). Genetic diversity in the simulated captive population (with breeding size *N* = 64 for 24 generations) was lower than that of the wild population (which declined from *N* = 1000 to *N* = 110 during this time). Hence, releasing more than two captive‐bred individuals every generation accelerates genomic erosion of the rescued wild population. On the other hand, the small size of the captive population also helped to purge some of its genetic load, particularly of severely deleterious mutations. This explains why the genetic load in the rescued population declines as more captive individuals are released (Figure 6a).

Our computer models reveal that a reintroduction strategy aimed at genetic rescue might need to trade off the benefits of reducing the genetic load and realised load against the loss of genetic diversity, adding to the recent literature concerning genetic rescue (Bell et al., 2019; Jackson et al., 2022; Pérez‐Pereira et al., 2022; Ralls et al., 2020; Robinson et al., 2021, 2023; Smeds & Ellegren, 2023). Maximizing the success of genetic rescue relies on finding the optimum number of captive individuals designated for release, which can be accomplished through genomics‐informed management and simulations. Genomics can also help to identify which individuals are likely to make the most valuable contribution to the wild gene pool, by either masking the realised load (see Speak et al., 2023) and/or by increasing diversity that has been lost from the wild. Our simulations also illustrate the power of purging of the genetic load in the small captive population, which is relevant to the debate about what size of source population is most suited for genetic rescue (i.e., a large or small source population, see, e.g., Ralls et al., 2020), and the ‘One Plan’ approach in conservation (Segelbacher et al., 2022). Whole‐genome sequence data can also be used to estimate long‐term *N*_*e*_, which can enable assessment of whether or not the ratio between *N*_*e*_ and census population size (*N*_*c*_) is inflated—such elevated *N*_*e*_/*N*_*c*_ ratios are indicative of continued genomic erosion (van Oosterhout, 2024; Wilder et al., 2023). With respect to our study species, the presence of a genetically diverse and diverged captive population might help to secure the long‐term viability of Arabia's last big cat.

## CONFLICT OF INTEREST STATEMENT

The authors declare no conflict of interest.

## ETHICS STATEMENT

Ethical approval for this research was received from the Research Ethics Committee of the School of Anthropology and Conservation (July 2017), University of Kent. Details available upon request.

## Supporting information


Data S1.

## Data Availability

The data that support the findings of this study are available upon request from the corresponding author.
