# Supplementary material for: Can genetic rescue help save Arabia's last big cat?
Source: Evol Appl. 2024 May 23;17(5):e13701. doi: 10.1111/eva.13701 (PMC11113348; doi:10.1111/eva.13701)
Supplement: Supplementary file 1 — Data S1. [file EVA-17-e13701-s001.docx]

**Supplementary Information**

***Study site***

Within the Dhofar mountains, the Jabal Qamar and Jabal Qara, are strongly influenced by the annual monsoon (mid-June to mid-September) and as a result a unique cloud forest, rich in endemic flora (Patzelt, 2015) is found on the plateaus and south-facing aspects. The third mountain, Jabal Samhan, has the highest elevation (1,760 meters above sea level [a.s.l]) but is hardly affected by the monsoon and is consequently mostly hyper-arid with sparse desert vegetation. The northern foothills of these mountains are known as the Nejd, and although leopards have been observed in this region (Al Hikmani et *al.,* 2015), the Nejd is considered to be on the periphery of core leopard habitat (Mazzolli *et al.*, 2017).

***Sampling information***

*Wild samples: scat, skin, and blood*

Scat was collected from Oman during targeted presence/absence and capture-recapture (camera trap) surveys, in four sampling areas; Jabal Samhan, Jabal Qara, Jabal Qamar and the Nejd (Figure 3a-c). To improve sampling success we conducted surveys along predefined transects, chosen to optimize detection based on information from previous camera trap and scat surveys in each sampling area (Figure 3a-c). Between 5^th^ January and 13^th^ April 2017 repeated surveys were conducted on multiple routes across Jabal Samhan (43 routes: 88 km, completed four times), Jabal Qamar (26 routes: 75 km, completed three times), Jabal Qara (16 routes: 46 km, completed twice), and the Nejd (17 routes, 49 km, completed twice) giving 102 routes covering 767 km. Opportunistic collection of scat from non-leopard surveys between December 2016 and April 2017 augmented our targeted scat surveys. Scats were dated, georeferenced, and collected and stored in plastic ziplock bags at room temperature.

Biological material was obtained from 15 wild Arabian leopards (mostly wild-killed between 1976 and 2008) comprising skin samples (n = 11) from the Omani Natural Heritage Museum (ONHM), Muscat, Oman; Harrison Institute, Sevenoaks, UK; Directorate General of Environment and Climate Affairs, Salalah, Oman; Directorate General of Agricultural and Livestock Research, Rumais, Muscat, Oman; and blood samples (n = 4) from the Breeding Centre for Endangered Arabian Wildlife in Sharjah, UAE (BCEAW); and co-author AJS (Table S1).

*Captive samples*

Samples of skin (n = 26), blood (n = 9) and scat (n = 3) from captive leopards were obtained from the Omani Wild Animals Breeding Centre, Muscat, Oman; Prince Saud Al-Faisal Wildlife Research Centre, Taif, Saudi Arabia; and BCEAW. Ten of the sampled leopards were wild-born in Yemen (Table S1).

*DNA collection from scat, tissue and blood*

Approximately 200 mg of dried scat powder was scraped from the outer surface of each scat into a 2 ml microcentrifuge tube using a sterilised razor blade and subsequent DNA extraction followed the manufacturer’s instructions.

DNA was extracted from skin, blood and bone samples using a Qiagen DNAeasy Blood and Tissue Kit (Qiagen, UK). The initial digestion approach to DNA extraction varied in terms of the quantity of initial material used for the DNA extraction depending on the type of source material. For blood samples, we used 100 µl of blood mixed with 100 µl of phosphate buffered saline (PBS) and 20 µl of proteinase K. For skin samples, pieces of skin < 25 gm were cut, then finely chopped using a clean razor blade and placed into a 2 ml microcentrifuge with 300 µl of ATL buffer and 25 µl of proteinase K. The solution was then vortexed and incubated overnight at 56°C on a mechanical mixer. Museum skin samples and any contemporary skin samples that were particularly dry were washed and soaked in PBS for 24 to 48 hours before extraction. Bone samples from which samples were to be subsequently taken were first cleaned with double-distilled water followed by 96% ethanol. The bones were then cleaved, crushed and ground using a cleaned hammer and Sabatier bow saw. The resulting powder (approximately 100 gm) was then placed in a 2 ml microcentrifuge tube with 320 µl of ATL buffer, 40 µl of proteinase K and 40 μl of 0.5 M EDTA and incubated overnight at 56°C. DNA obtained from scat and skin samples was eluted into 100 µl of elution buffer; 200 µl for blood samples and 50 µl for bone samples respectively. For downstream PCR amplification, DNA from blood and skin samples was further diluted to an appropriate concentration using purified water. As the procedure of DNA extraction from scat and museum samples can be susceptible to contamination, we used aerosol barrier pipette tips when pipetting and conducted DNA extraction steps inside a pre-sterilized UV fume hood.

### DNA extraction and species identification using mtDNA

We performed PCR amplification in reaction volumes of 10 µl containing 5 µl MyTaq HS Red Mix (Bioline), 1.6 µl dH_2_O, 0.2 µl (0.2 µM) of each forward and reverse primer, 1µl BSA (0.01 μg/μL) (Bovine Serum Albumin, New England Biolabs Inc.) and 2 µl of DNA. PCR cycling conditions consisted of an initial hot start of 95°C for 8 min followed by 35 cycles of 94°C for 30 s, 50°C for 1 m and 72°C for 1 s, and a final incubation period of 10 min at 72°C. To reduce the risk of contamination, PCR preparation and DNA extraction were performed in separate laboratories at the Durrell Institute of Conservation and Ecology (DICE), University of Kent. All PCRs included a negative control. PCR products were initially visualised on 2% agarose gels using electrophoresis to check for amplification and to monitor for signs of contamination in negative controls. PCR products that indicated DNA originating from leopard scat were then purified and sequenced using a 3730X analyser (Macrogen, Amsterdam, Netherlands). The resulting forward and reverse sequences were edited and aligned using Jalview v2 (Waterhouse *et al.,* 2009). Individual consensus sequences were then searched for using BLAST (NCBI); samples from which sequences aligned with the single Arabian leopard NADH5 sequence (GenBank accession: AY035279) were assumed to be leopard; non-leopard DNA samples were excluded from further analyses.

*PCR conditions used for PCR sexing loci*

PCR reactions (10 µl volume) contained 5µl Qiagen multiplex PCR buffer mix (Qiagen Inc.), 0.2 µl (0.2 µM) fluoro-labelled forward primer (Eurofins Genomics), 0.2 µl (0.2 µM) reverse primer, 0.5 µl (0.005 μg/μL) BSA, 0.5 µl PCR anti-inhibitor and 3 µl of template DNA. The PCR cycling conditions for all multiplexes consisted of an initial denaturation of 95°C for 15 min, 45 cycles of denaturation (94°C for 30 s), annealing (Ta ranges from 54°C to 58°C for 90 s), extension (72°C for 90 s), and a final extension of 10 min at 72°C.

### Assessment of genotypes

To reduce errors associated with degraded DNA we genotyped each sample at least three times and determined consensus genotypes using the multiple-tubes approach (Taberlet *et al*., 2002). Samples that amplified fewer than four loci were discarded from further analysis. We accepted a genotype to be true if repeated genotypes matched 100% across all loci at least twice, otherwise the sample was excluded. Allelic dropout and false alleles were identified using GIMLET v1.3.3 (Valiere, 2002). Scoring errors and null alleles were identified using Microchecker (Van Oosterhout *et al.,* 2004). Deviation from Hardy-Weinberg equilibrium (HWE) and linkage disequilibrium (LD) was assessed using Genepop 4.7 (Raymond & Rousset, 1995) with sequential Bonferroni correction (Rice, 1989) applied for multiple LD tests. Genetic diversity metrics were calculated using GenAlEx v6.5 (Peakall & Smouse, 2006). We measured allelic richness and accounted for uneven sample size using the diveRsity (Keenan *et al.,* 2013) package in R (R Core Team, 2018).

***Analyses of levels of genetic diversity***

GenAlEx v6.5 was used to quantify the extent of spatial genetic differentiation (F_ST_) between populations and to perform a spatial autocorrelation analysis. The spatial autocorrelation analysis was performed on the genotypes of 36 individuals at eight loci specifying a total of 11 distance classes of size 20. The analysis was run for 999 permutations with 1000 bootstraps. To infer fine scale population structure we used GENELAND v1.0.7 (Guillot *et al.,* 2005a) to examine signals of genetic structure within the dataset comprising leopards from the Dhofar mountain ranges, following two steps as per Guillot *et al.* (2005a). GENELAND uses individual multi-locus genotypes together with their geographic locations to infer the number of populations and identify any genetic discontinuity within these populations (Guillot *et al.,* 2005b). GENELAND analysis is considered to provide superior estimates of the number of clusters as it takes account of the geographic location of each sample, and is considered more robust in instances where there is relatively weak genetic structure (Basto *et al.*, 2016). We first applied 10 independent runs with 500,000 MCMC iterations and a burn-in of 100 under the spatial model, specifying uncorrelated allele frequency assuming unknown K. To generate a map of the distribution of each cluster, and accurate individual assignment, we repeated the analysis but treated the number of clusters as known, using a previously determined number from step one (K=3).

For comparisons of heterozygosity, we applied unbiased expected heterozygosity (uHe) as it is appropriate for datasets that are likely to contain samples from close relatives or inbred individuals (DeGiorgio & Rosenberg, 2009). To test for differences in genetic diversity between wild and captive populations, regions, or time periods, we performed one-way ANOVAs. We then subjected the data to an analysis of molecular variance (AMOVA) using GenAlEx 6.5 to test for population differentiation.

***Estimation of temporal change in Ne***

TMVP uses the collection date of each sample and allele frequencies to estimate N_e_ at the time of collection of the oldest sample (N_a_= historical), and at the time of collection of the most recent sample (N_0_=contemporary) while accounting for uneven sample size across both sampling periods, and loci. We used a generation time of four years (Dutta *et al.*, 2013) with rectangular priors of 0–1000 for historical and contemporary N_e_. Given the census population size of Jabal Samhan leopards is considered to have been < 200 in the 1970s (2.30 leopard/100km^2^; this study) with an approximate historical range size at that time of 4000 km^2^ (HAH. *unpubl*.), we estimated the current population size to be ~92 leopards, and subsequently re-ran the model using priors of 0–200. The oldest and most recent Jabal Samhan samples date from 1977 and 2017 respectively.

***Estimation of density and population size***

*Molecular DNA (scat) surveys*

To estimate leopard population density, we used scat samples (n= 270) collected between 5^th^ January and 13^th^ April 2017 only, thereby meeting the SECR timeframe parameter of ~3 months to establish closed population size in large carnivores (Karanth *et al.,* 2002). Sixty-nine of these samples produced genotypes which amplified for more than four loci, and were then used to determine the number and density of leopards in Dhofar.

*Camera trap surveys*

Between 5^th^ January and 13^th^ April 2017 while on scat-collection surveys in the Jabal Samhan study region, we simultaneously deployed 42 camera trap (Bushnell Trophy Cam: Model 119456) stations (Figure 3a-c). To increase detection probability (e.g. detections of the same individual at multiple locations) stations were spaced approximately two kilometres apart. Cameras operated continuously over the deployment period and were programmed to take three photographs at one second intervals when triggered. Identification of individual leopards is possible through their unique spot patterns. Therefore to increase the probability of both captures and photo-identifiable spot patterns from images of the left and/or right flanks, two cameras were placed approximately opposite each other on either side of the route at each station. Stations received maintenance checks and new SD cards and batteries every two weeks.

We were unable to deploy camera traps while performing scat surveys in Jabal Qara, Jabal Qamar, and the Nejd. To compensate, we used camera trap data collected between 20^th^ September and 15^th^ December 2013, and 10^th^ April and 20^th^ July 2014 from deployments in Jabal Qara (13 stations) and Jabal Qamar (20 stations) (Figure 3b-c). Deployment follows that previously described, with the exception that Jabal Qara stations were spaced 3-10 km apart. No alternative camera trap data are available for the Nejd. Captures for independent density estimates were insufficient (*N* = < 10) for SECR, but we subsequently used these data to determine the minimum number of leopards in each region and to support identification from scat surveys.

Individual leopards were visually identified from photographs based on unique rosette patterns such as the shape, size, and formation of the spots on the flanks (Figure S3), in addition to other distinctive markings (Spalton *et al.*, 2006). Genitalia were used to determine sex, and reference photographs from previous surveys were used to aid identification. Each identified individual was given a unique reference number. Blurred or otherwise poorly detailed photographs were disregarded.

To avoid overinflation of zero values, which could lead to low estimates of detection probability, camera data were grouped into eight occasions (each occasion consisting of eight days) while genotype ‘recaptures’ from scats were assigned to either two occasions (Jabal Qamar) or three occasions (Jabal Samhan).

For SERC analysis we assumed a Poisson distribution of leopards and used the half-normal detection model while detector type was specified as proximity (Efford et al., 2009). We used the buffer function in SECR to infer the appropriate buffer to measure effective survey area, but initial results included areas of non-leopard habitat (open desert, water bodies, settlements) which may bias results (Efford, 2011). We therefore used ArcGIS v10.5 to create a map of suitable habitat based on leopard distribution from Spalton & Al Hikmani (2014) (Supplementary Information, Figure S1) and ran SECR analysis based on maps of potential habitat using constant detection probability (lambda = λ: λ0~1) and spatial movement models (sigma = σ: σ~1). We also ran individual heterogeneity models (2–class finite mixture: λ0~h2 σ~1, λ0~1 σ~h2, λ0~h2 σ~h2; see Efford, 2019, for details and descriptions of models used) for each data set. Since leopard movement varies between males and females, sex was included as a covariate in all models. Models were ranked based on the Akaike information criterion (AIC) using AICwt and delta AICc to correct for small sample sizes (Burnham & Anderson, 2002). Models with delta ≤ 2 were considered to have more support. As no single model had a weight (AICwt) of 0.9 final density was computed using the model averaging function in SECR.

**Habitat corridors to reconnect Dhofar populations**

Our study finds evidence of genetic structure between the leopards of western, central and eastern Dhofar. Given the evidence for population subdivision and restricted gene flow between Arabian leopard populations, we recommend urgent identification and subsequent protection of potential corridors to facilitate reconnection between leopard sub-populations in the Dhofar mountains. For example, conservation authorities should consider implementing conservation measures to ensure the continuity of leopard movement between western Jabal Qara and the Nejd. Although based on limited sample size the leopard populations in these two regions are shown to have gene flow between them, and they also exhibit relatively high levels of genetic diversity in comparison to other regions in Dhofar; i.e. Jabal Samhan, Jabal Qamar. We also advocate that consideration is given to creating and establishing wildlife crossings such as under- and over-passes along the main roads in Jabal Qara. Wildlife crossing structures have been shown to enable dispersal among several taxa including bears (Sawaya *et al.,* 2014), wolves (Shepherd & Whittington, 2006) and cougar *Puma concolor* (Gloyne & Clevenger, 2001). The creation and protection of habitat connectivity through such corridors could promote dispersal and geneflow across existing barriers to leopard dispersal, facilitating the reduction of inbreeding (and inbreeding depression), and thereby maximising population persistence. Such initiatives will also be beneficial to other terrestrial species of the region that live alongside the Arabian leopard such as the Arabian wolf and Nubian ibex.

**Table S1.** Details of Arabian leopard biological samples collected from known and captive leopards for genetic diversity study. **MECA**=Ministry of Environment and Climate Affairs, **MAF**=Ministry of Agriculture and Fisheries, **OWABC** = Oman Wild Animal Breeding Centre, **ONHM**= Oman Natural History Museum, **HI**= Harrison Institute, Sevenoak (UK), **BCEAW**= Breeding Centre for Endangered Arabian Wildlife in Sharjah (UAE), **PSAWRC** = Prince Saud Al-Faisal Wildlife Research Center in Taif (KSA).

| **ID** | **Origin** | **Status** | **Year** | **Sample type** | | | **Obtained from** |
| --- | --- | --- | --- | --- | --- | --- | --- |
|  |  |  |  | Scat | Blood | Skin |  |
| 1 | Musandam/Oman | Wild-killed | 1976 |  |  | x | HI |
| 2 | Samhan/Oman | Wild-killed | 1977 |  |  | x | HI |
| 3 | Samhan/Oman | Wild-killed | 1977 |  |  | x | HI |
| 4 | Musandam/Oman | Wild-killed | 1979 |  |  | x | HI |
| 5 | Samhan/Oman | Wild-killed | 1979 |  |  | x | HI |
| 6 | Samhan/Oman | Wild- killed | 1982 |  |  | x | ONHM |
| 7 | Samhan/ Oman | Wild caught | 1985 |  | x |  | BCEAW |
| 8 | Samhan /Oman | Wild- caught | 1985 |  |  | x | ONHM |
| 9 | Samhan/ Oman | Wild-caught | 1989 |  |  | x | ONHM |
| 10 | Samhan /Oman | Wild -caught | 1997 |  |  | x | ONHM |
| 11 | Samhan/ Oman | Wild -caught | 2002 |  | x |  | Dr Andrew Spalton |
| 12 | Samhan/ Oman | Wild -caught | 2002 |  | x |  | Dr Andrew Spalton |
| 13 | Samhan /Oman | Wild- killed | 2003 |  |  | x | MECA, Oman |
| 14 | Samhan/Oman | Wild- caught | 2006 |  | x |  | Dr Andrew Spalton |
| 15 | Salalah /Oman | Wild -killed | 2008 |  |  | x | MAF, Oman |
| 16 | Captive born | Captive | 2016 |  |  | x | OWABC |
| 17 | Captive born | Captive | 2016 |  |  | x | OWABC |
| 18 | Captive born | Captive | 2016 |  |  | x | OWABC |
| 19 | Captive born | Captive | 2017 |  |  | x | BCEAW |
| 20 | Captive born | Captive | 2017 |  |  | x | BCEAW |
| 21 | Captive born | Captive | 2017 |  |  | x | BCEAW |
| 22 | Captive born | Captive | 2017 |  |  | x | BCEAW |
| 23 | Captive born | Captive | 2017 |  |  | x | BCEAW |
| 24 | Captive born | Captive | 2017 |  |  | x | BCEAW |
| 25 | Captive born | Captive | 2017 |  |  | x | BCEAW |
| 26 | Captive born | Captive | 2017 |  |  | x | BCEAW |
| 27 | Captive born | Captive | 2017 |  |  | x | BCEAW |
| 28 | Captive born | Captive | 2017 |  |  | x | BCEAW |
| 29 | Captive born | Captive | 2017 |  |  | x | BCEAW |
| 30 | Captive born | Captive | 2017 |  |  | x | BCEAW |
| 31 | Captive born | Captive | 2017 |  |  | x | BCEAW |
| 32 | Captive born | Captive | 2017 |  |  | x | BCEAW |
| 33 | Captive born | Captive | 2017 |  |  | x | BCEAW |
| 34 | Captive born | Captive | 2017 |  |  | x | BCEAW |
| 35 | Captive born | Captive | 2017 |  |  | x | BCEAW |
| 36 | Captive born | Captive | 2016 |  | x |  | PSAWRC |
| 37 | Captive born | Captive | 2016 |  | x |  | PSAWRC |
| 38 | Captive born | Captive | 2016 |  | x |  | PSAWRC |
| 39 | Captive born | Captive | 2016 |  | x |  | PSAWRC |
| 40 | Captive born | Captive | 2016 |  | x |  | PSAWRC |
| 41 | Captive born | Captive | 2016 |  |  | x | PSAWRC |
| 42 | Captive born | Captive | 2016 | x |  |  | PSAWRC |
| 43 | Captive born | Captive | 2016 | x |  |  | PSAWRC |
| 44 | Wild born Yemen | Captive | 2016 |  | x |  | PSAWRC |
| 45 | Wild born Yemen | Captive | 2016 |  | x |  | PSAWRC |
| 46 | Wild born Yemen | Captive | 2016 |  | x |  | PSAWRC |
| 47 | Wild born Yemen | Captive | 2016 |  |  | x | PSAWRC |
| 48 | Wild born Yemen | Captive | 2016 |  |  | x | PSAWRC |
| 49 | Wild born Yemen | Captive | 2016 | x |  |  | PSAWRC |
| 50 | Wild born Yemen | Captive | 2017 |  |  | x | BCEAW |
| 51 | Wild born Yemen | Captive | 2017 |  |  | x | BCEAW |
| 52 | Wild born Yemen | Captive | 2017 |  |  | x | BCEAW |
| 53 | Wild born Yemen | Captive | 2016 |  | x |  | OWABC |

**Table S2.** Details of microsatellites multiplexes used in this study.

| Multiplex | Number of markers | Temp | FAM | HEX | NED |
| --- | --- | --- | --- | --- | --- |
| **1** | 4 | 58 | FCA52 | FCA126  6HDZ635 | FCA90 |
| **2** | 5 | 55 | FCA193 | FCA149 | FCA309  FCA77  FCA96 |
| **3** | 7 | 56 | FCA123  FCA391 | FCA672  FCA229  FCA220  6HDZ859 | ZN |
| **4** | 4 | 58 | FCA105 | 6HDZ700 | FCA205  6HDZ610 |
| **5** | 6 | 54 | FCA304  FCA26 | FCA441  AM | FCA310  FCA45 |
| **6** | 7 | 58 | FCA075  FCA453  6HDZ817 | FCA628  FCA224  6HDZ7 | 6HDZ317 |
| **7** | 4 | 58 | F41 | 6HDZ64 | FCA279 |

Locus AM was also amplified separately using a lower annealing temperature (T_a_ 52 C).

**Table S3.** Partitioning of Arabian leopard genotype data set for genetic diversity measurements and comparisons between different populations and regions, including temporal comparison in Jabal Samhan population (Objective 1 of this study. The analyses were based on samples that amplified for at least 5 loci).

| Partitioning of the genotype dataset and comparisons | Sample size |
| --- | --- |
| (a) Comparison of genetic diversity between wild leopards from Oman and captive leopards (including wild- born leopards from Yemen) | n=45 Oman  n=33; captive |
| (b) Comparison of genetic diversity between wild leopards from Oman and wild- born leopards from Yemen | n =45; Oman  n=8; Yemen |
| (c) Comparison of contemporary genetic diversity between regions in Dhofar: Jabal Samhan, Jabal Qara, Jabal Qamar, Nejd | n=17; Jabal Samhan  n=5; Jabal Qara  n=11; Jabal Qamar  n=3; Nejd |
| (d) Temporal measures of genetic diversity in Jabal Samhan population for three periods: 1976-1985, 1997-2006, 2012-2017 | n=4; 1976-1985 period  n=5; 1997-2006 period  n=17; 2012-2017 period |

**Figure S1.** Map of potential habitat of the Arabian leopard in the Dhofar mountains based on leopard presence data from camera traps and GPS collars (Spalton & Al Hikmani, 2014).


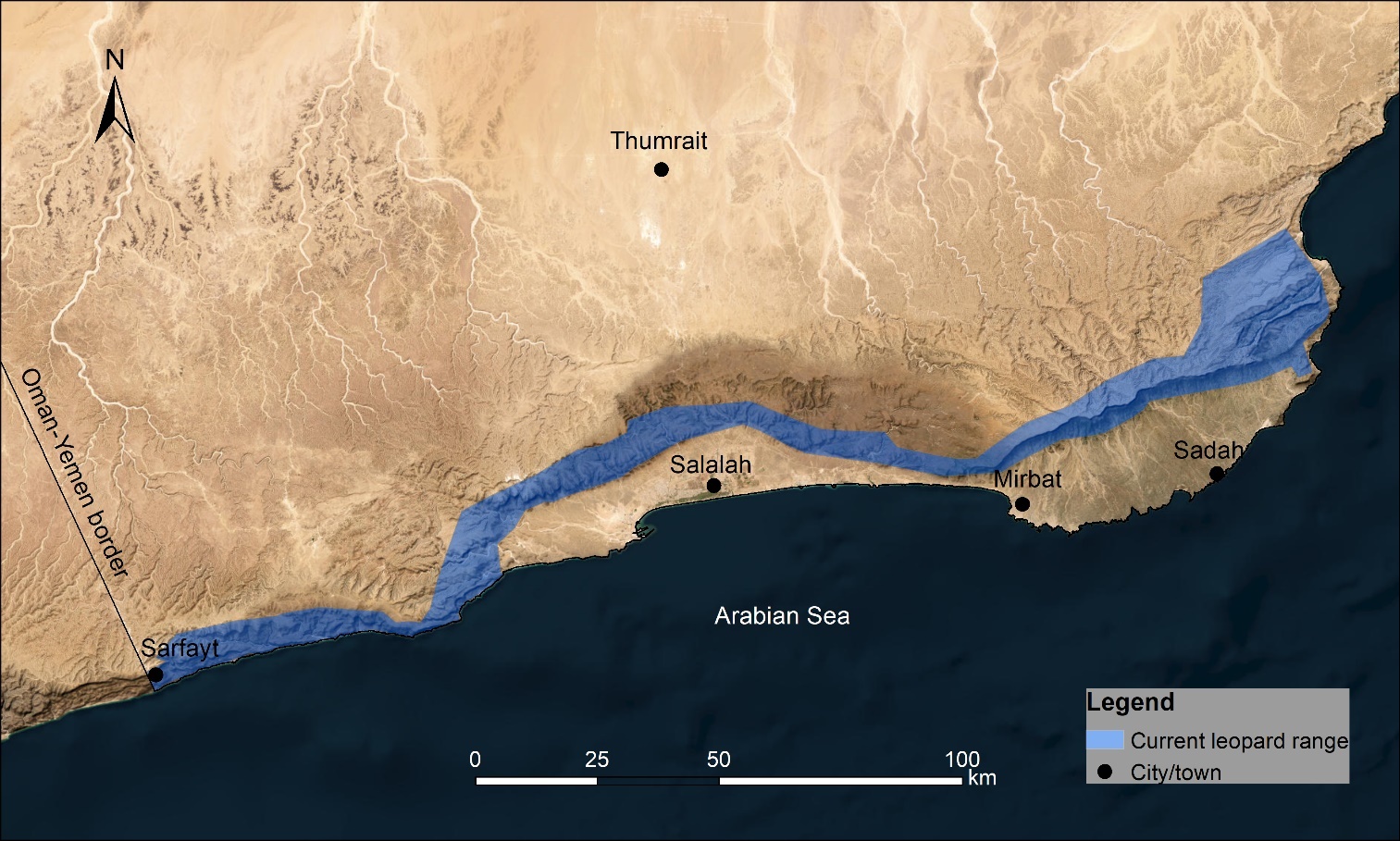


**Table S4.** Vortex parameters used for the simulations of growth or decline of the wild Arabian leopard population following alternative scenarios of supplementation via reintroduction.

| **Input Variable** | **Value** | |
| --- | --- | --- |
| Number of iterations | 1000 |  |
| Number of years | 100 |  |
| Duration year in days | 365 |  |
| Extinction definition | one sex |  |
| Number of populations | 2 (wild, captive) |  |
| Order of events: EV, Breed, Mortality, Age, Disperse, Harvest, Suppl. |  |  |
| *Lethal Equivalents* | 6.29/13.58 |  |
| *Percentage inbreeding due to LE* | 50% |  |
| *Environmental correlation between reproduction and survival* | 0.5 |  |
| *Environmental correlation among populations* | Yes |  |
|  |  | |
| ***System*** | Polygynous | |
| *Age of first offspring female* | 3 |  |
| *Age of first offspring male* | 4 |  |
| *Max lifespan* | 20 |  |
| *Max age female reproduction* | 15 |  |
| *Max age male reproduction* | 15 |  |
| *Max number broods per year* | 1 |  |
| *Max progeny per brood* | 3 |  |
| *Sex ratio at birth* | 50 |  |
| *% adult female breeding* | 50 |  |
| *SD in % breeding due to EV* | 10 |  |
|  |  |  |
|  | **Wild** | **Captive** |
| Mortality 0-1 | 50 | 32 |
| SD | 10 | 5 |
| Mortality 1-2 | 20 | 10 |
| SD | 3 | 3 |
| Mortality 2+ | 10 | 10 |
| SD | 3 | 3 |
| Initial populaiton size | 51 | 62 |

***SLiM simulations to estimate genetic load, realised load, mean fitness and neutral nucleotide diversity***

In the model, fitness (w) is a function of the realised load expressed in lethal equivalents (LEs), (Bertorelle et al. 2022): 𝑤=𝑒−^𝑅𝑒𝑎𝑙𝑖𝑠𝑒𝑑 𝐿𝑜𝑎𝑑^. The simulated populations consisted of diploid individuals with males and females in equal sex ratio. Their genomes comprised an exome composed of 9 pairs of autosomes, each with 1000 genes of 1500 nucleotides, similar to the study of Beichmann et al. (2023). Free recombination occurs between autosomes. The recombination rate within and between genes was set at r=1e-9 and r=1e-3, respectively. Ancestral populations were generated using a two-stage burn-in to attain a mutation-selection-drift equilibrium. First, a neutral burn-in without mutations (Mu=0) was run for 990,000 generations to ensure complete coalescence. Coalescence is necessary when measuring neutral nucleotide diversity which is performed after simulations have finished by overlaying neutral mutation on tree sequence data to improve computational performance. In the second stage of the burn-in, the mutation rate was set to produce a masked load of ~6 lethal equivalents (LEs) at equilibrium. The second stage of the burn-in ran 10,000 generations to complete. This genetic load is comparable to that used in our Vortex simulations, and consistent with previous studies. For example, O’Grady et al. (2006) estimated 12 diploid LEs (6 haploid LEs) across the life-history of the species in a meta-analysis. The census population size for both stages of the burn-in was set at N=21,000, which is consistent with the estimated ancestral population size of the Arabian leopard (Al Hikmani et al. 2023).

Deleterious mutations were sampled from previously modelled stable populations. The HS distribution is displayed in Figure S2. Deleterious mutations were introduced from the second burn-in stage onwards at a rate of 2.4e-8. Neutral mutations were added overlain to trees sequences that were output from the simulation at a rate of 1.6-8 and used to assess neutral diversity and segregating sites.

**Figure S2.** The HS distribution of the mutations used in the SLiM simulations.


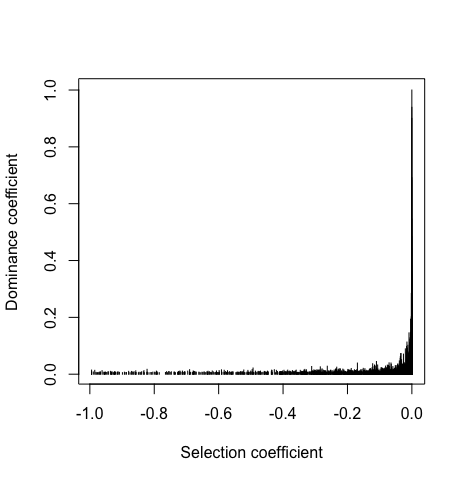


In our SLiM model, the population experienced a linear decline from N=21,000 to N=1000 over 2000 generations. We thus assumed that the wild population declined over the past 10,000 years, and that the generation time was 5 years. At the end of the decline, 64 individuals (32 male, 32 female) were separated from the wild population to form the captive population. The wild and captive populations were reproductively isolated for a further 24 generations, which represents the start of the 20th Century to the present day. During this period, the captive population size remained at a constant population size of N=64, while the wild population declined further from N=1000 to N=110 at a linear rate of decline. Finally, we simulated 100 years (or 20 generations) into the future (from the year 2024 to 2124), examining the impact of different rescue scenarios. In particular, we simulated the release of 0, 2, 4, 6, or 8 captive individuals (with a 1:1 sex ratio) every generation (5 years). During this time, the captive population size remained constant at N=64 whilst the size of the wild population varied randomly between N=32–79 (the 95% CIs of the population mean estimate of N=51). Neutral nucleotide diversity, genetic load, realised load and fitness were recorded after 100 years (20 generations). Simulations were run 100 times for each setting.

Information on the distribution of fitness effects, dominance distribution and their effect on the fitness of individuals is given in Figure S2.

**Figure S3**. An illustration of the distribution of fitness effects, dominance distribution, and fitness distribution at the conclusion of the generation of the burn-in populations. The distribution of fitness effects and dominance distribution were generated by randomly sampling 10,000 mutations from the burnt-in populations. The fitness distribution represents the fitnesses of all individuals from 10 simulations.


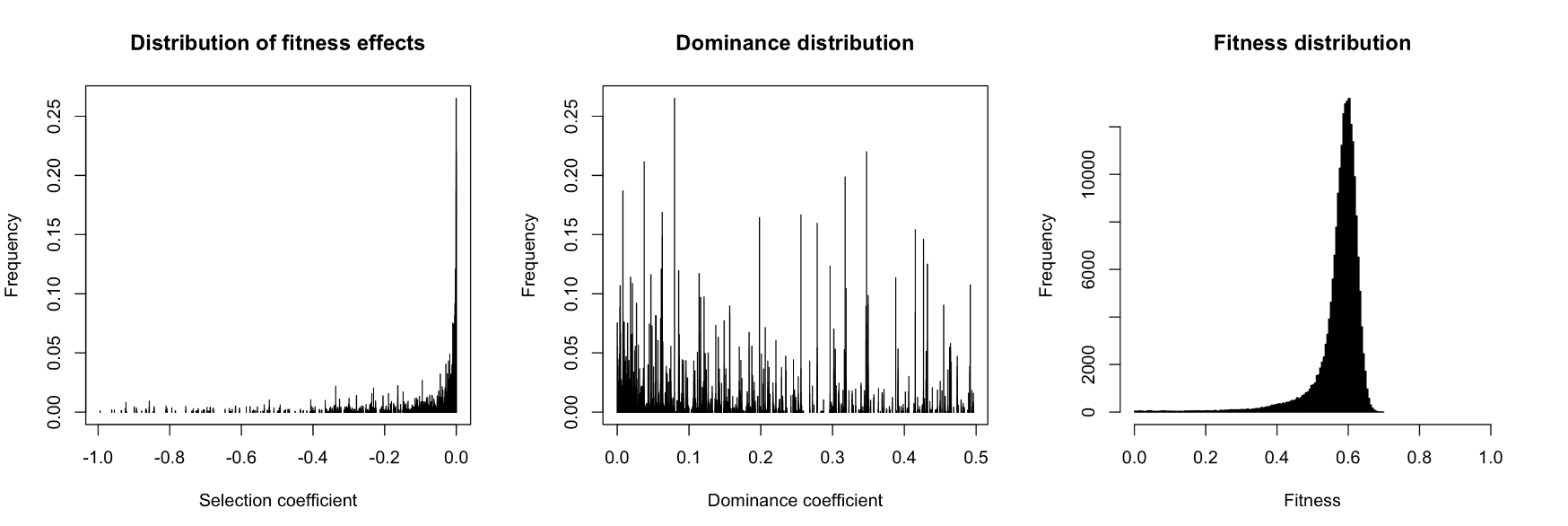


**Table S5**. Summary of Arabian leopard individuals that were genotyped from each population.

| Population | Total samples | Samples genotyped for at least 5 loci |
| --- | --- | --- |
| Oman | 51 | 45 |
| Yemen | 10 | 8 |
| Captive-born | 28 | 25 |

**Table S6.** Summary of scat samples collected, screened to exclude non-target species, and then genotyped from each of the sampled regions in Dhofar, Oman between 12th January 2012 and 6th April 2017.

| Regions | Samples collected from the field | Samples genetically verified to be leopard | Samples genotyped for at least 5 loci | No. of individual leopards |
| --- | --- | --- | --- | --- |
| Jabal Samhan | 191 | 113 | 70 | 17 |
| Jabal Qara | 95 | 11 | 9 | 5 |
| Jabal Qamar | 161 | 32 | 26 | 11 |
| Nejd | 30 | 5 | 4 | 3 |
| Total | 477 | 161 | 109 | 36 |

**Table S7**. Characteristics of the microsatellite markers amplified in the Arabian leopard and their characteristic in other leopard subspecies.

| Locus/subspecies | This study | Amur leopard | Indian leopard | African leopard |
| --- | --- | --- | --- | --- |
| F41 | Polymorphic | Not used | Polymorphic | Not used |
| FCA90 | Polymorphic | Polymorphic | Polymorphic | Not used |
| FCA105 | Polymorphic | Polymorphic | Not used | Polymorphic |
| FCA126 | Polymorphic | Not used | Polymorphic | Polymorphic |
| FCA279 | Polymorphic | Not used | Polymorphic | Not used |
| 6HDZ89 | Polymorphic | Not used | Not used | Not used |
| 6HDZ635 | Polymorphic | Not used | Not used | Not used |
| 6HDZ700 | Polymorphic | Not used | Not used | Not used |
| F52 | Monomorphic | Not used | Polymorphic | Not used |
| FCA075 | Monomorphic | Polymorphic | Not used | Monomorphic |
| FCA453 | Monomorphic | Not used | Polymorphic | Polymorphic |
| 6HDZ817 | Monomorphic | Not used | Not used | Not used |
| FCA628 | Monomorphic | Not used | Polymorphic | Polymorphic |
| FCA224 | Monomorphic | Polymorphic | Not used | Monomorphic |
| FCA309 | Monomorphic | Not used | Polymorphic | Not used |
| 6HDZ610 | Monomorphic | Not used | Not used | Not used |
| FCA310 | Monomorphic | Not used | Polymorphic | Polymorphic |
| 6HDZ64 | Monomorphic | Not used | Not used | Not used |

**Figure S4.** Location of scat survey routes (blue lines), camera traps and individual leopards identified from scat and camera survey techniques in **(a)** Jabal Samhan, (**b)** Jabal Qara (survey routes in the north represent the Nejd study region) and (**c)** Jabal Qamar and Nejd.


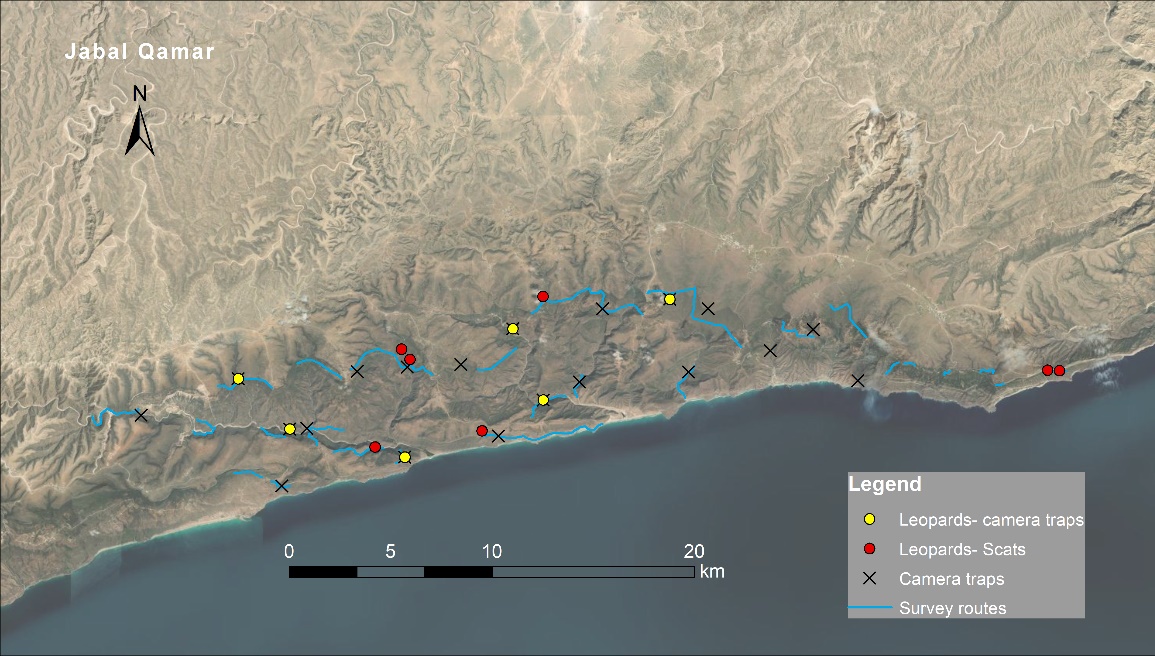

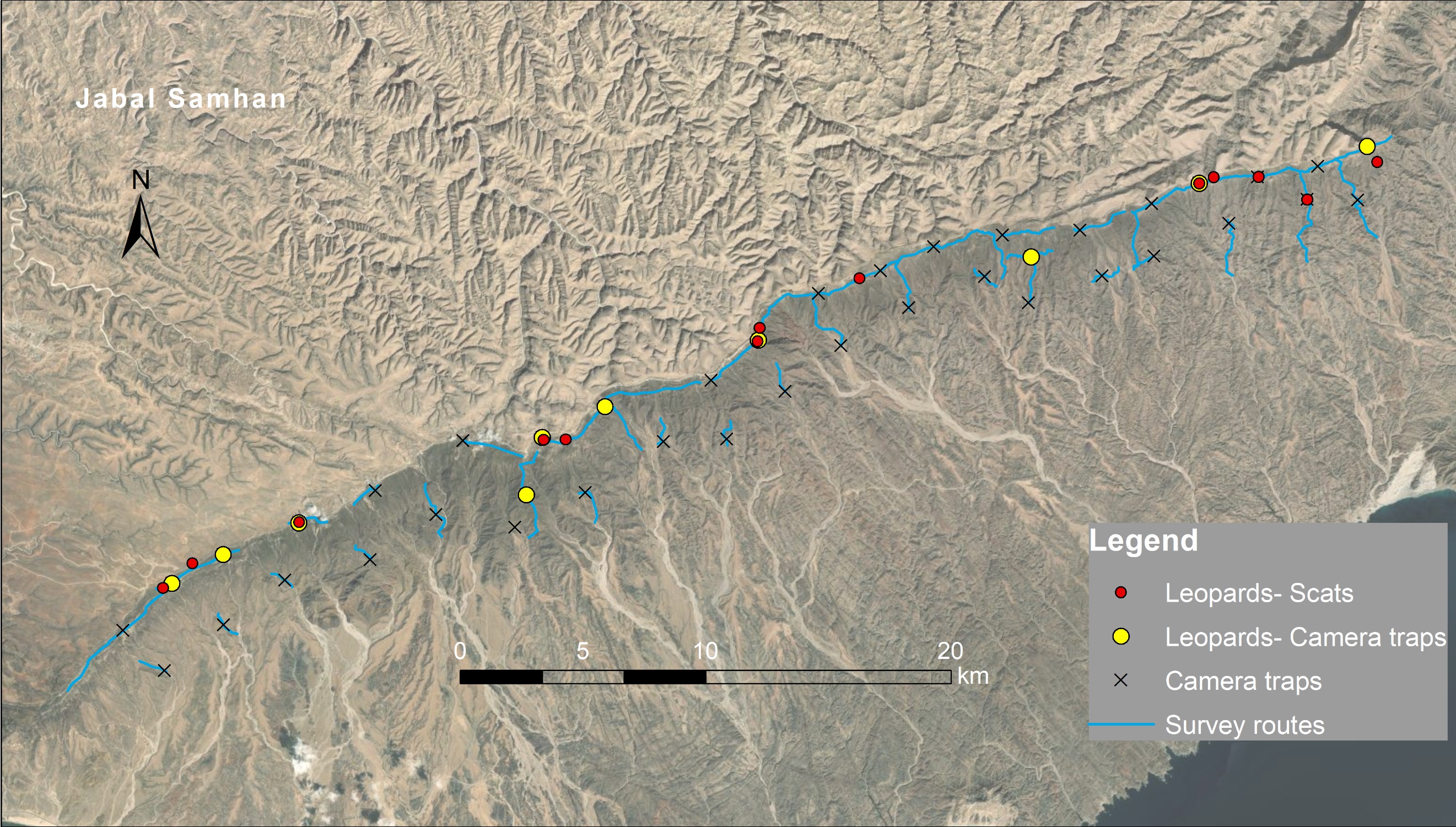

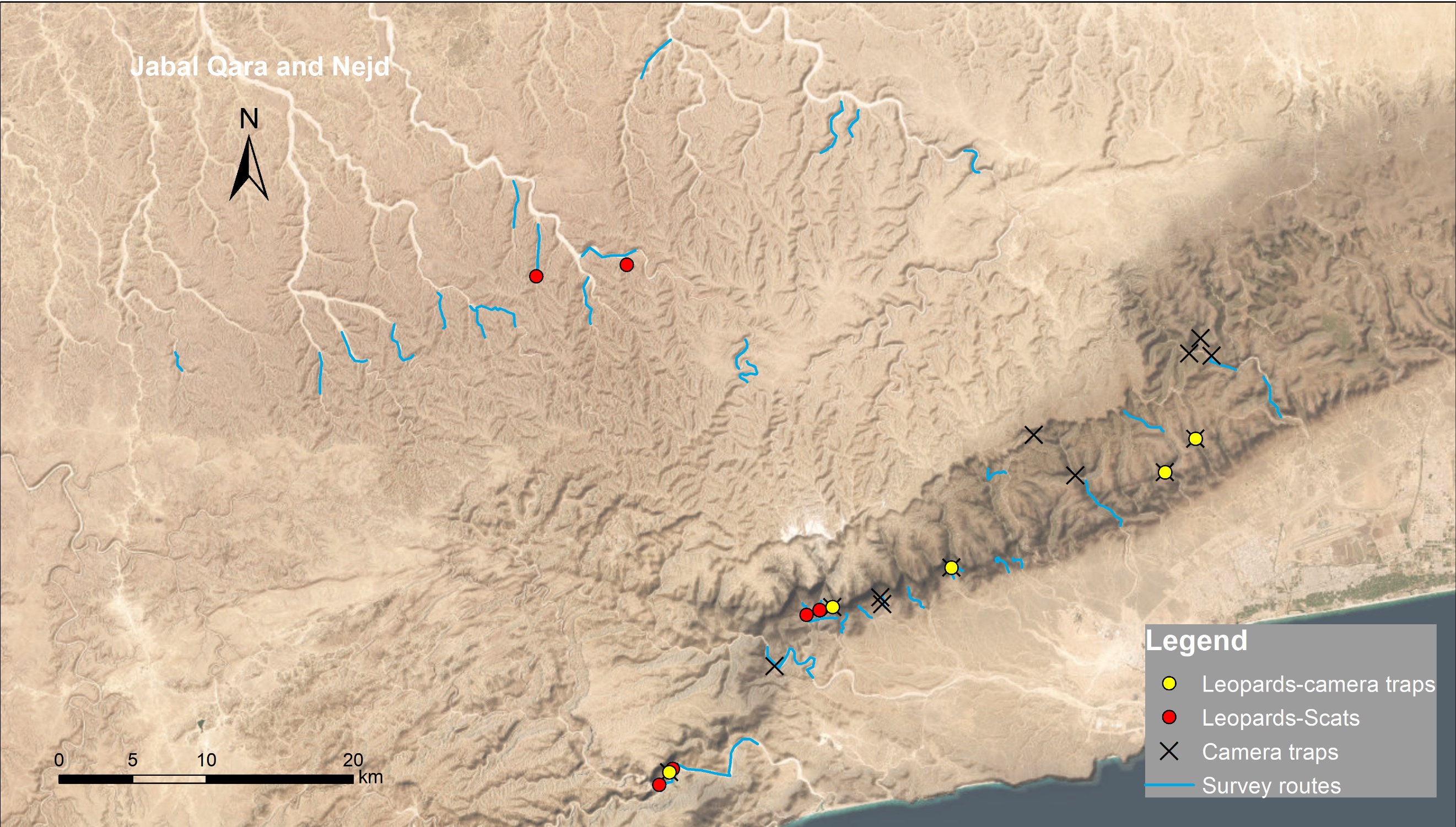


**Figure S5.** (a) Plot and regression line of Mantel pairwise data for the full Arabian leopard data set based on genetic and geographic pairwise distance (r = 0.13964, P (rxy-rand > = rxy-data) = 0.001). (b) plot of extent of correlation (r) across geographic distance (end point of class). U, upper 95% CI, L, lower 95% CI.

**(b)**

**(a)**

**Figure S6.** Detection history of leopard generated in SECR modelling. The red crosses are camera trap/scat sites and coloured circles are individual leopards. Locations of symbols relate to the relative geographic position of cameras/scats derived from the original latitude/longitude coordinates.
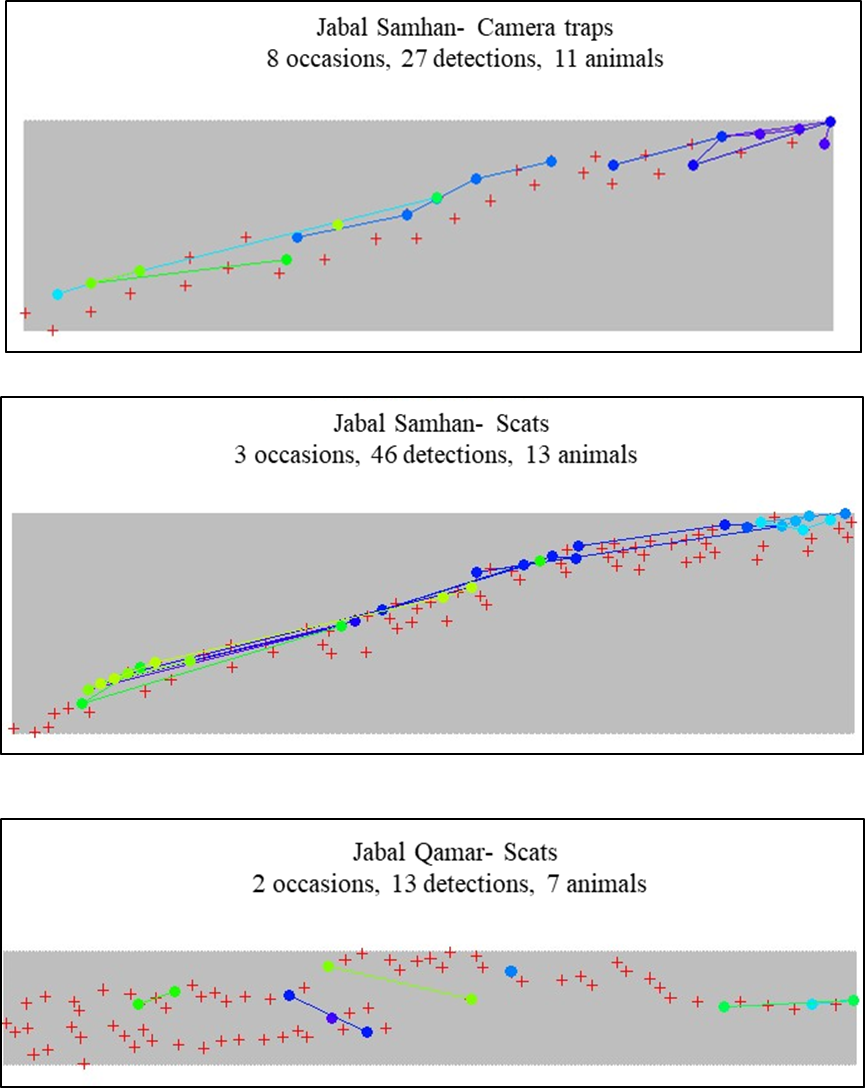


**Figure S7**. Number of individual leopards detected across the Dhofar mountains based on camera trap surveys and genotyping of scats from field surveys.

Number of individuals

**Table S8.** Model selection results from Arabian leopard density estimates using photographic and genetic capture-recapture data from Dhofar in the program SECR using half normal detection function. Lambda0 is the capture probability at home range center. Sigma is the spatial scale parameter of capture function. ℎ2 is the 2-class finite mixture probability for heterogeneity. dAIC is Akaike’s information criterion adjusted for small sample size. AICwt represents Akaike weight. Bold indicates the model that fit the data and has strong support. When there is support for more than one model, density is estimated using the model averaging function in SECR (see text for details).

| Region | Model | No parameters | dAIC | AICwt |
| --- | --- | --- | --- | --- |
| Samhan_camera2017 |  |  |  |  |
|  |  |  |  |  |
| **Model_Samhan_camera_2** | **lambda0~h2 sigma~1** | **5** | **0** | **0.5086** |
| **Model_Samhan_camera_3** | **lambda0~h2 sigma~h2** | **6** | **0.316** | **0.4342** |
| Model_Samhan_camera_0 | lambda0~1 sigma~1 | 4 | 7.241 | 0.0136 |
| Model_Samhan_camera_1 | lambda0~1 sigma~h2 | 5 | 8.204 | 0.0084 |
| Samhan _Scat2017 |  |  |  |  |
| **Model_Samhan_scat_0** | **lambda0~1 sigma~1** | **4** | **0** | **0.5084** |
| **Model_Samhan_scat_2** | **lambda0~h2 sigma~1** | **5** | **1.682** | **0.2193** |
| **Model_Samhan_scat_1** | **lambda0~1 sigma~h2** | **5** | **1.961** | **0.1907** |
| Model_Samhan_scat_3 | lambda0~h2 sigma~h2 | 6 | 3.658 | 0.0816 |
| Qamar_scat2017 |  |  |  |  |
| **Model_Qamar_scat_0** | **lambda0~1 sigma~1** | **4** | **0** | **0.4349** |
| **Model_Qamar_scat_2** | **lambda0~h2 sigma~1** | **5** | **0.934** | **0.2726** |
| **Model_Qamar_scat_1** | **lambda0~1 sigma~h2** | **5** | **1.65** | **0.1906** |
| Model_Qamar_scat_3 | lambda0~h2 sigma~h2 | 6 | 2.901 | 0.102 |
| Overall scats (Samhan and Qamar) | |  |  |  |
| **Model_scat_Dhofar_0** | **lambda0~1 sigma~1** | **4** | **0** | **0.7354** |
| Model_scat_Dhofar_2 | lambda0~h2 sigma~1 | 5 | 3.502 | 0.1277 |
| Model_scat_Dhofar_1 | lambda0~1 sigma~h2 | 5 | 3.609 | 0.121 |
| Model_scat_Dhofar_3 | lambda0~h2 sigma~h2 | 6 | 7.659 | 0.016 |

**Figure S**8**.** The location of the Dhofar mountains in Oman, home to the Arabian leopard, and the Wada’a region in northwest Yemen from where the wild born ‘Yemen’ leopards are thought to have
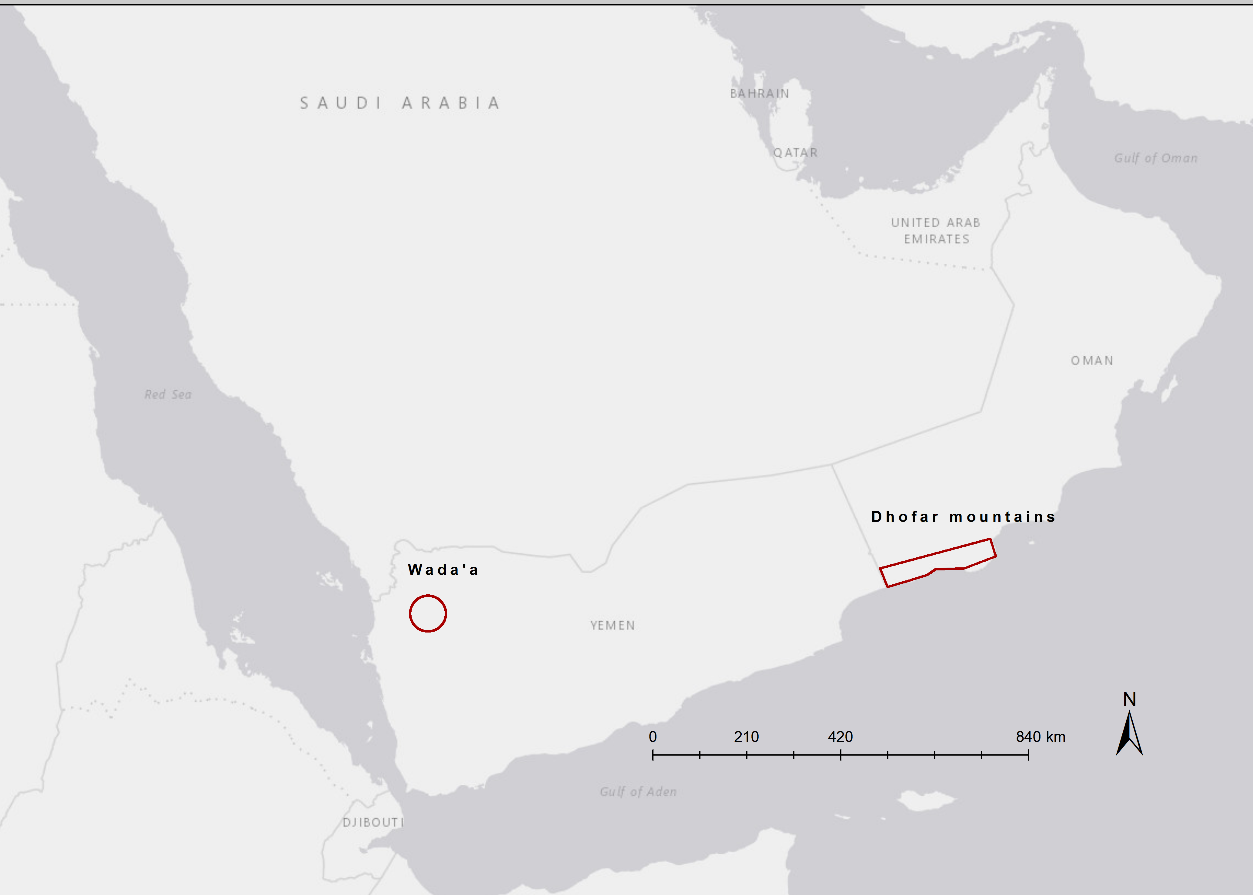
been sourced for the captive population (Al Jumaily et al., 2006).

**Figure S9**. Estimates of genetic differentiation (F_ST_) between the main populations of Arabian leopards of Dhofar. Dashed lines delineate between the geographically distinct regions.


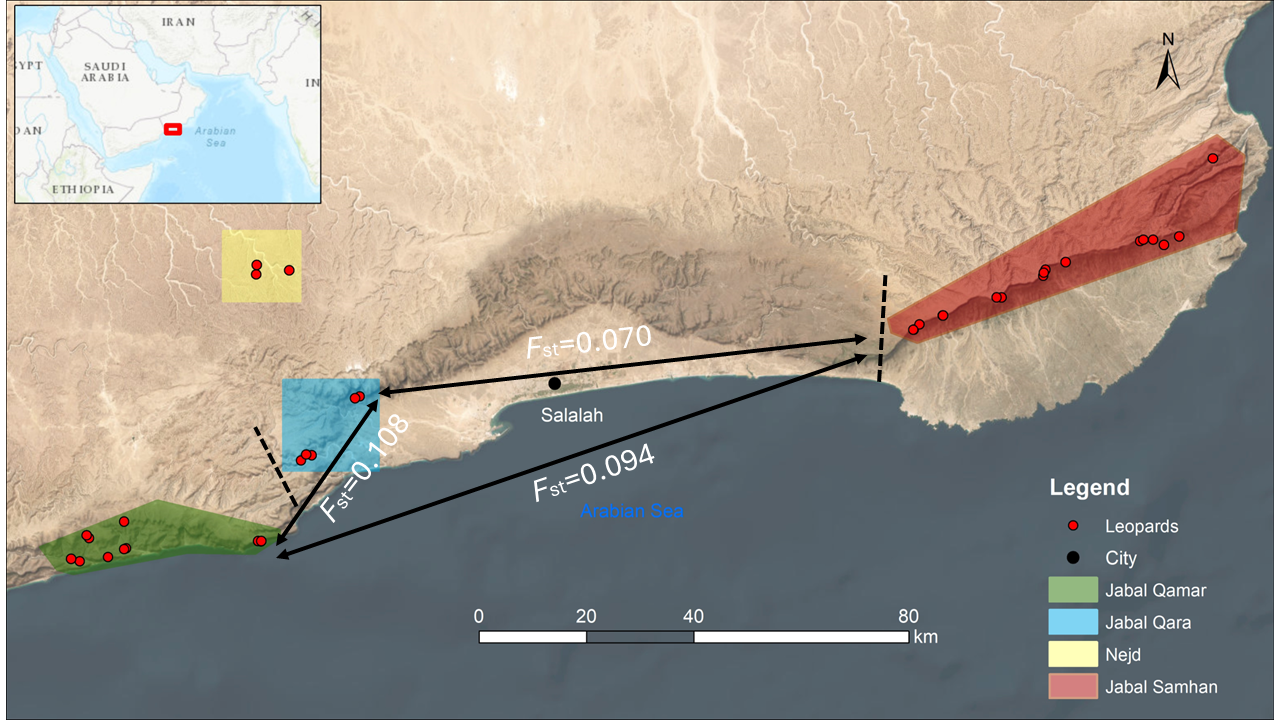


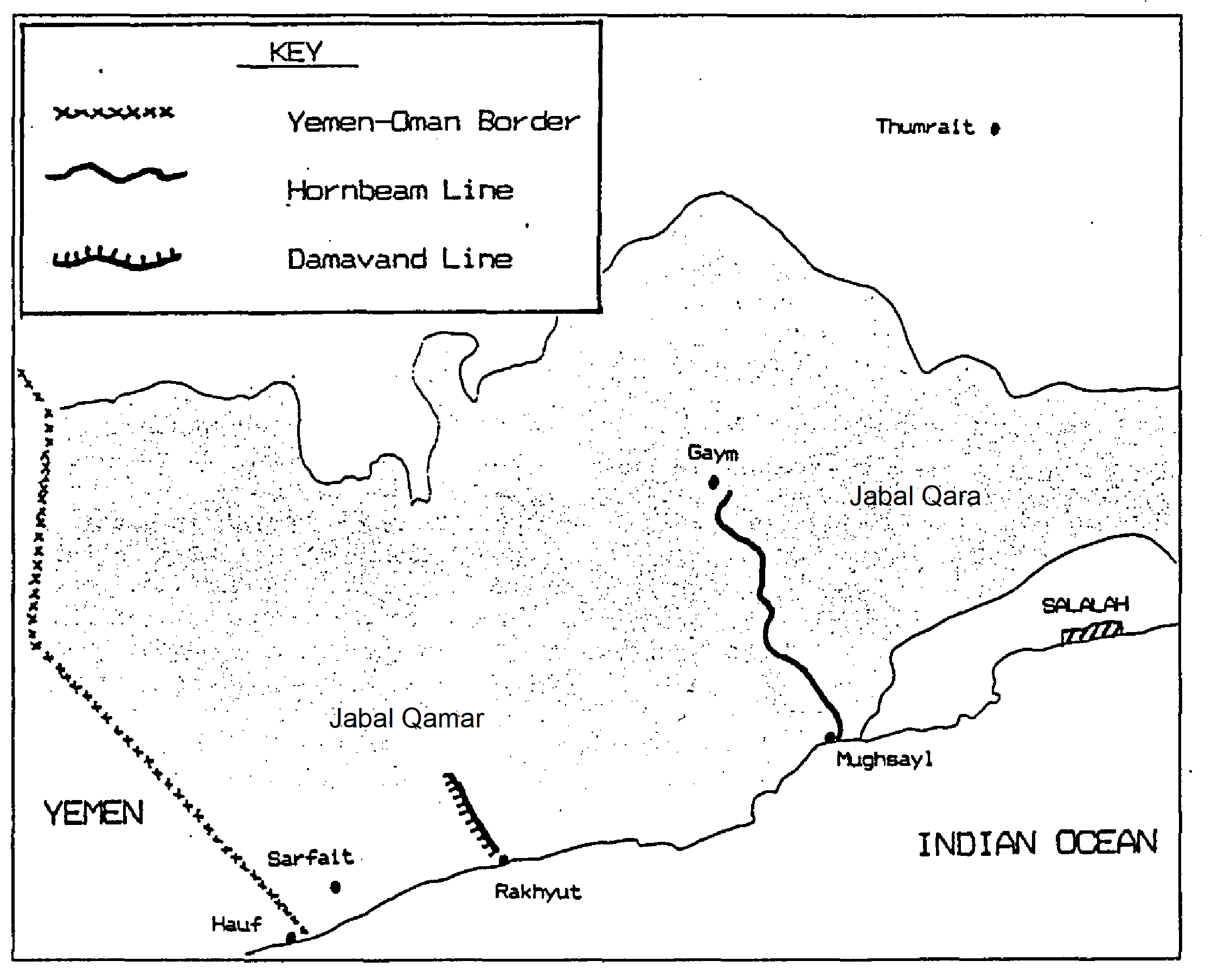
**Figure S10:** The position of the Hornbeam defence line in Jabal Qara, extracted from Tuas (1988).


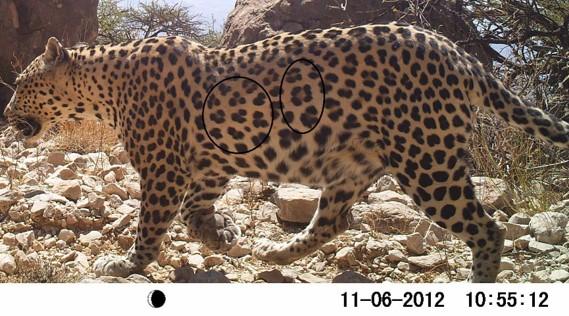
**Figure S**11**.** Example of individually identifiable leopards based on their spot patterns. Top and bottom photos show the same individual male leopard that was recorded in different locations (in this instance in Jabal Samhan), almost one year apart.


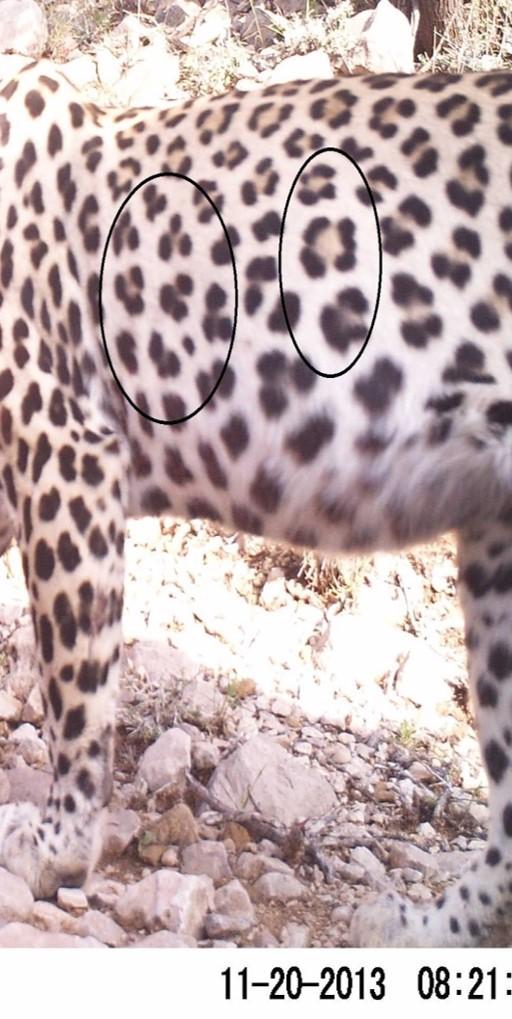


**Cited reference**

Al Hikmani, H., Zaabanoot, N. & Zaabanoot, A. (2015). Camera trapping of Arabian leopard in the Nejd region of Dhofar Mountains*. Cat News*, 62, 32.

Barson, N. J., Cable, J & van Oosterhout, C. (2009). Population genetic analysis of microsatellite variation of guppies (*Poecilia reticulata*) in Trinidad and Tobago: Eevidence for a dynamic source–sink metapopulation structure, founder events and population bottlenecks. *Journal of Evolutionary Biology*, 22(3), 485–- 497.

Basto, M. P., Santos-Reis, M., Simoes, L., Grilo, C., Cardoso, L., Cortes, H., Bruford, M. W. & Fernandes, C. (2016). Assessing genetic structure in common but ecologically distinct carnivores: The stone marten and red fox. *PLoS One*,11, e0145165.

Beichman, A.C., Kalhori, P., Kyriazis, C.C., DeVries, A.A., Nigenda-Morales, S., Heckel, G., Schramm, Y., Moreno-Estrada, A., Kennett, D.J., Hylkema, M. & Bodkin, J. (2023).

Genomic analyses reveal range-wide devastation of sea otter populations*. Molecular*

*Ecology*, 32(2), 281–-298.

Bertorelle, G., Raffini, F., Bosse, M. Bortoluzzi, C., Iannucci, A, , Trucchi E., Morales H. E., van

Oosterhout C.*et al*. (2022). Genetic load: Genomic estimates and applications in non-model animals. *Nature Reviews Genetics,* 23 (8), 492–503.

Burnham, K. P. & Anderson, D. R. (2002). Model selection and multimodel inference: A practical

information-theoretic approach, 2nd edn. Springer-Verlag, New York. New York

Springer.

DeGiorgio, M. & Rosenberg, N. A. (2009). An unbiased estimator of gene diversity in samples containing related individuals. *Molecular Biology and Evolution*, 26, 501–512.

Dutta, T., Sharma, S., Maldonado, J. E., Wood, T. C., Panwar, H. S. & Seidensticker, J. (2013). Gene flow and demographic history of leopards (*Panthera pardus)* in the central Indian highlands. *Evolutionary Applications*, 6, 949–959.

Dutta, T., Sharma, S., Maldonado, J. E., Wood, T. C., Panwar, H. S., & Seidensticker, J. (2013b). Fine‐scale population genetic structure in a wide‐ranging carnivore, the leopard (Panthera pardus fusca) in central India. Diversity and Distributions, 19, 760–771.

Efford, M. G. (2011). Estimation of population density by spatially explicit capture-recapture

analysis of data from area searches. *Ecology*, 92, 2202–2207.

Efford, M. G. (2019). Finite mixture models in secr 3.2 [https://www.otago.ac.nz/density/pdfs/secr- finitemixtures.pdf](https://www.otago.ac.nz/density/pdfs/secr-%20finitemixtures.pdf)

Gloyne, C. C. & Clevenger, A. P. (2001). Cougar *Puma concolor* use of wildlife crossing structures

on the trans-Canada highway in Banff National Park, Alberta. *Wildlife Biology*, 7, 117–124.

Guillot, G., Estoup, A., Mortier, F. & Cosson, J. F. (2005a). A spatial statistical model for landscape genetics. *Genetics,* 170, 1261–1280

Guillot, G., Mortier, F. & Estoup, A. (2005b). GENELAND: A computer package for landscape genetics. *Molecular Ecology Notes*, 5, 712–715.

Karanth, U.K., Kumar, NS. & Nichols, J.D. (2002). Field surveys: Estimating absolute densities of tigers using capture-recapture sampling In: Karanth U.K., Nichols, JD. (eds*). Monitoring of tigers and their prey. A manual for researchers, managers and conservationists in tropical Asia (*pp*.* 111– 120). Centre for Wildlife Studies.

Keenan, K., McGinnity, P., Cross, T.F., Crozier, W.W. & Prodöhl, P. A. (2013). diveRsity: An R package for the estimation and exploration of population genetics parameters and their associated errors. *Methods in Ecology and Evolution*, 4, 782–788.

Mazzolli, M., Haag, T., Lippert, B. G., Eizirik, E., Hammer, M. L. A. & Al Hikmani, K. (2017). Multiple methods increase detection of large and medium-sized mammals: working with volunteers in south-eastern Oman. *Oryx*, *51*, 290–297.

O'’Grady J. J., Brook, B.W., Reed, D.H.,. Ballou, J. D., Tonkyn, D.W. & Frankham, R. (2006).

Realistic levels of inbreeding depression strongly affect extinction risk in wildpopulations*. Biological Conservation,* 133(1), 42–51.

Patzelt, A. (2015). Synopsis of the flora and vegetation of Oman, with special emphasis on patterns of plant endemism. In: Abhandlungen der Braunschweigischen Wissenschaftlichen Gesellschaft, pp. 282–317.

Peakall, R. & Smouse, P. (2006). GENALEX 6: Genetic analysis in excel. Population genetic software for teaching and research. *Molecular Ecology Resources*, 6, 288–295.

Raymond, M. & Rousset, F. (1995). GENEPOP v1.2: Population genetics software for exact tests and ecumenicism. *Journal of Heredity*, 86, 248–249.

Rice, W. R. (1989). Analyzing tables of statistical tests*. Evolution*, 43, 223–225.

Sawaya, M. A., Kalinowski, S. T. & Clevenger, A. P. (2014). Genetic connectivity for two bear

species at wildlife crossing structures in Banff National Park. Proceedings of the

Royal Society B: Biological Sciences, 281.

Shepherd, B. & Whittington, J. (2006). Response of wolves to corridor restoration and human use

management*. Ecology and Society*, 11 (2): [online] <http://www.ecologyandsociety.org/vol11/iss2/art1/>.

Spalton, A. & Al Hikmani, H. (2014). The Arabian leopards of Oman. Stacey International,

UK.

Spalton, J. A., Al Hikmani, H. M., Willis, D. & Bait Said, A. S. (2006). Critically endangered

Arabian leopards *Panthera pardus nimr* persist in the Jabal Samhan nature reserve, Oman. *Oryx*, 40, 287–294.

Taberlet, P. (2002). Reliable genotyping of samples with very low DNA quantities using PCR. *Nucleic Acids Research*, 24, 3189–3194.

Valiere, N. (2002). GIMLET: Aa computer program for analysing genetic individual identification data. *Molecular Ecology Notes*, 3, 377–379.

Van Oosterhout, C., Hutchinson, W. F., Wills, D. P. M. & Shipley, P. (2004). MICRO-CHECKER: Software for identifying and correcting genotyping errors in microsatellite data. *Molecular Ecology Notes,* 4, 535–538

Waterhouse, A. M., Procter, J. B., Martin, D. M. A., Clamp, M. & Barton, G. J. (2009). Jalview version 2. A multiple sequence alignment editor and analysis workbench. Bioinformatics, 25, 1189–1191.
